# Systems Biology and Structure-Based In Silico Evaluations of Quercetin–Psychobiotic Interactions for the Management of Depression

**DOI:** 10.3390/ph19091366

**Published:** 2026-08-28

**Authors:** Samridhi Kurl, Neeraj Mittal, Rajwinder Kaur, Balakumar Chandrasekaran, Gurpreet Kaur

**Affiliations:** 1 Department of Pharmaceutical Sciences and Drug Research, Punjabi University Patiala, Patiala 147002, Punjab, India; samridhi_rs24@pbi.ac.in; 2 Chitkara College of Pharmacy, Chitkara University, Rajpura 140401, Punjab, India; mittal.neeraj@chitkara.edu.in (N.M.); rajwinder.kaur@chitkara.edu.in (R.K.); 3 Department of Pharmaceutical Sciences, Faculty of Pharmacy, Zarqa University, Zarqa 13111, Jordan

**Keywords:** structure-based drug design, network pharmacology, genome-scale metabolic modelling, molecular docking, molecular dynamics simulation, gut–brain axis, psychobiotics

## Abstract

**Background/Objectives:** Psychobiotics are live microorganisms that, when administered in appropriate amounts, can enhance mental health. Through the production of neuroactive metabolites, regulation of immune responses, and interactions along the gut–brain axis, they modulate host neurophysiology. However, significant strain-specific variability exists in their metabolic capabilities, colonization potential, and functional interactions, necessitating a systematic approach for psychobiotic strain selection. **Methods**: In the present study, an integrated in silico systems biology approach combining comparative genomics, genome-scale metabolic modeling, network pharmacology, molecular docking, molecular dynamics, and ADMET analysis was employed to computationally evaluate the therapeutic potential and molecular interactions of a quercetin-psychobiotic co-delivery system for managing depression by modulating the gut–brain axis. **Results**: Comparative genomic analysis of candidate psychobiotic strains from the genera *Bifidobacterium* and *Lactobacillus* predicted the presence of key functional genes involved in neuroactive metabolite production, immune modulation, oxidative stress tolerance, and adhesion. The presence of acetate-producing pathways alongside the absence of classical butyrate biosynthesis genes indicated an indirect contribution to short-chain fatty acid production via microbial cross-feeding interactions. Genome-scale metabolic Modeling under the conditions of quercetin supplementation predicted a significant inter-strain variation in terms of predicted biomass flux, nutrient utilization, and metabolic adaptability. Results of molecular docking showed that the binding energies were in the range of −5.854 to −9.489 kcal/mol. **Conclusions**: Collectively, these in silico findings provided a computational basis for the selection of psychobiotic strains and predicted that a combination of psychobiotics and quercetin could present a promising strategy for the modulation of the gut–brain axis.

## 1. Introduction

Depression is a widespread and complex mental disorder that affects over 332 million people globally [1]. It has a high prevalence exceeding 7%, with an associated suicide rate of approximately 10% [2,3]. With the emergence of the COVID-19 pandemic, an approximate increase of 25% in the cases of depression has been further observed [4]. As per reports, till 2020, 45.7 million people were affected by depression in India only [5]. Antidepressant medications, such as selective serotonin reuptake inhibitors (SSRIs) and monoamine oxidase (MAO) inhibitors, are the current treatment strategies for the management of depression [6,7]. However, these medications do not provide a definitive cure. They only provide symptomatic relief and are also associated with various adverse effects, such as drug tolerance, drug dependence, nausea, vomiting, and diarrhea [8]. Furthermore, 35% of patients suffer from Treatment-Resistant Depression (TRD). Also, vulnerable population groups such as children, adolescents, and pregnant women have restricted access to conventional antidepressants [7]. Therefore, there is an urgent need to develop safer and effective alternatives that can minimize the associated side effects and improve therapeutic outcomes.

A significant association between the gut microbiota and depression has been demonstrated by recent research, showing the important role of the gut–brain axis in mental health. The pathophysiology of depression has been linked with alterations in the composition of gut microbiota, commonly referred to as “microbial dysbiosis”. Decreased levels of beneficial bacteria such as *Coprococcus*, *Faecalibacterium*, and *Sutterella*, and imbalances in the population of bacteria, such as an increased *Bacteroidetes*-to-*Firmicutes* (B/F) ratio, contribute to neuroinflammation and altered production of neurotransmitters [9]. Thus, gut microbiota modulation has emerged as a promising strategy for the management of depression.

Psychobiotics, which are a subset of probiotics, are emerging as a safer alternative for the treatment of depression due to their potential to demonstrate a positive impact on mental well-being. The term “Psychobiotics” was introduced in 2013 and is defined as “live microorganisms that, when administered in appropriate amounts, can enhance mental health” [10]. Various pathways are involved in the communication between these beneficial bacteria and the central nervous system, such as modulation of tryptophan metabolism, increased levels of brain-derived neurotrophic factor (BDNF) and serotonin, reduced inflammation, and stimulation of the vagus nerve [11,12]. For patients who experience adverse effects from conventional antidepressant medications or exhibit poor therapeutic response, psychobiotics are very beneficial [13]. It has been demonstrated that specific strains of *Lactobacillus* and *Bifidobacterium* modulate neurotransmitter levels, regulate immune responses, enhance intestinal barrier integrity, and influence stress-related pathways in the host [11,12]. However, the mechanistic understanding of strain-specific effects and their interactions with host pathways remains limited, despite growing evidence supporting their therapeutic potential in the management of depression.

In addition to microbial-based therapies, auxiliary treatment strategies involving dietary phytoconstituents are also gaining considerable attention. Quercetin was selected for the present study because it is a naturally occurring flavonol and it shows significant antidepressant potential due to its known neuroprotective and anti-inflammatory effects [2,14]. Quercetin has also been found to play a key role in treating mental disorders. It helps in enhancing gut health by modulating gut microbiota dysbiosis, restoring the structural and functional properties of the gut barrier, and supporting immune/mast cell stabilization [15]. It exerts a prebiotic effect by promoting the growth of beneficial bacteria, such as *Lactobacillus* and *Bifidobacterium*, thereby enhancing the production of short-chain fatty acids (SCFAs), which are essential for maintaining gut health [16,17]. Despite these promising attributes, the combined or synergistic effects of quercetin with psychobiotic strains remain largely underexplored.

The selection of an effective psychobiotic-prebiotic combination remains a major challenge due to the strain-specific nature and the complexity of microbial populations in the gut ecosystem. Traditional approaches for psychobiotic selection are mostly observational and often fail to take into consideration the inter-strain variability and critical differences in metabolic profile, host interaction profile, and colonization potential of the psychobiotics. Similarly, the identification of a suitable prebiotic that can stimulate the growth of psychobiotics is equally challenging due to diverse microbial metabolic pathways and cross-feeding interactions in the host gut microbiome [11].

To address these challenges, in silico approaches offer a more systematic and rational strategy for the selection of optimum psychobiotic–prebiotic combinations. Given the highly complex and dynamic nature of the gut–brain axis, in silico approaches in combination with integrative system biology provide a powerful framework for elucidating multi-layered biological interactions among the host genes, microbial metabolites, and metabolic pathways [18]. Thus, in the present study, an integrated in silico systems biology approach was used for the selection of potential psychobiotic strains and to explore the mechanistic basis of quercetin–psychobiotic-mediated gut–brain axis modulation in depression. The study encompasses the genomic characterization of selected psychobiotic strains to identify the most efficient candidates, followed by reconstruction and analysis of genome-scale metabolic models, and evaluation of metabolic interactions under defined conditions. This is followed by pathway enrichment and network pharmacology analyses that identify key host targets and signaling pathways, followed by molecular docking and molecular dynamics simulations. Finally, ADMET profiling is conducted to assess pharmacokinetic properties and safety.

## 2. Results

### 2.1. Genomic Analysis of Candidate Psychobiotic Strains for Psychobiotic Strain Selection

#### 2.1.1. Retrieval and General Features of Genomes

The genomic sequences of 12 candidate psychobiotic strains (*B. breve UBBR01*, *B. breve M16V*, *B. adolescentis 150*, *L. rhamnosus UBLR58*, *L. helveticus IDCC3801*, *L. plantarum UBLP40*, *L. plantarum NK151*, *L. plantarum 90sk*, *L. acidophilus LA5*, *L. plantarum PS128*, *L. helveticus R0052*, *and L. plantarum WLPL04*) were successfully retrieved from the NCBI Genome Database in both GenBank and FASTA formats. The genome sizes ranged from 2.2 Mb to 3.3 Mb. The GC content varied between 34–60%, and the number of predicted coding sequences (CDS) per strain was in the range of 1700 to 3100. Table 1 highlights the genomic characteristics of all the candidate psychobiotic strains.

#### 2.1.2. Functional Genome Annotation

**Figure 1:**
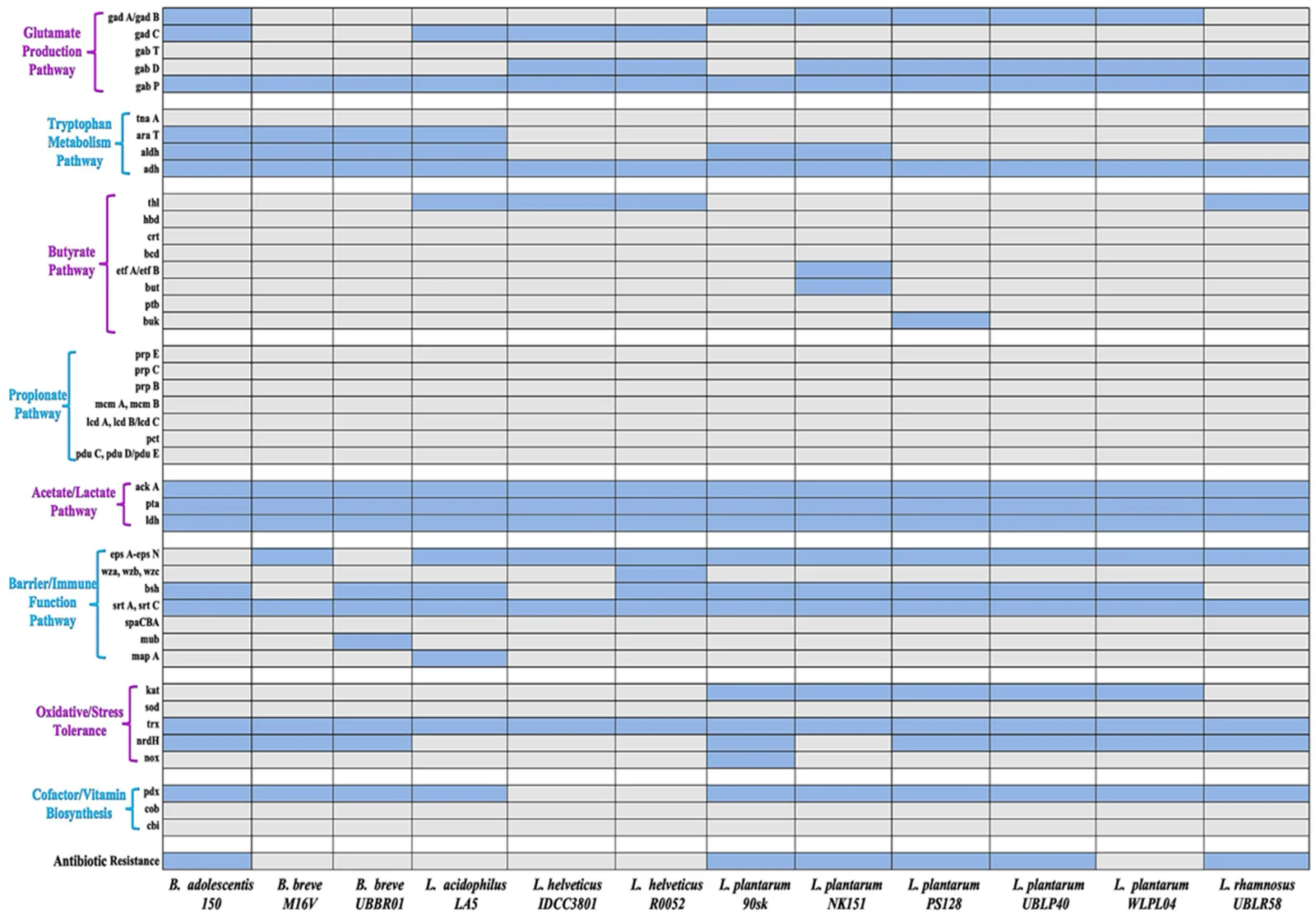
Heatmap representing the functional gene distribution across candidate psychobiotic strains.

Subsystem-level categorization of the candidate psychobiotic strains was obtained by functional genome annotation using RAST. The results obtained were organized systematically into major subsystems associated with psychobiotic-related traits, as shown in Figure 1. Rows represent the functional genes grouped into different biological pathways, whereas columns represent the candidate psychobiotic strains. Blue color indicates the presence of genes, and grey color indicates the absence.

Neuroactive Metabolite Pathways. Genes involved in the production of neuroactive metabolites were widely distributed across all candidate strains. Notably, genes involved in the production of glutamate, particularly the glutamate decarboxylase system (gadA, gadB, gadC), were present in all strains of *Lactobacillus plantarum* (*90sk*, *NK151*, *PS128*, *UBLP40*, and *WLPL04*) and in *Bifidobacterium adolescentis 150*. Genes encoding GABA transporters (gabP) were also detected across all strains, whereas the genes associated with the degradation of GABA (gadT and gadD) were absent across all strains. In contrast, the genes involved in the metabolism of tryptophan, leading to the formation of serotonin precursors, were found to be present across all the strains.

Production of Short-Chain Fatty Acids (SCFAs). Core genes involved in the production of acetate (ackA and pta) were consistently present across all strains, whereas the classical butyrate-producing genes (buk and but) required for the direct production of butyrate were absent in all genomes.

Barrier/Immune Function. Various genes associated with the interaction of the mucosal surface, modulation of the immune system, and gut barrier function were found across all strains, with a major distribution in the species of *Lactobacillus*. These include the genes involved in biosynthesis of exopolysaccharides (epsA–epsN), the export proteins (wzx, wzy, wze or wza, wzb, wzc), and the adhesin-related genes including mub, mapA, srtA, srtC, and spaCBA. Furthermore, the bile salt hydrolase (bsh) gene was also detected in the majority of strains.

Oxidative/Stress Tolerance. All the candidate strains harbored genes associated with oxidative stress tolerance. The antioxidant enzymes like catalase (kat) and superoxide dismutase (sod) were present in *L. plantarum* and *L. rhamnosus*, whereas they were absent in *Bifidobacteria*, consistent with their anaerobic nature. Redox-balancing genes (trx, nrdH, nox) were equally distributed in both genera.

Cofactor/Vitamin Biosynthesis. Genes involved in the biosynthesis of vitamins and cofactors were detected across all the strains and with notable genus-level variations. The genes associated with the biosynthesis of vitamin B6 (pdx) were present in all the strains of *Bifidobacteria* and *Lactobacillus* except in *L. helveticus*. The genes related to cobalamin (vitamin B12) biosynthesis (cob and cbi) were absent in both genera.

### 2.2. Antibiotic Resistance and Virulence Gene Profiling

All the strains were screened against the comprehensive antibiotic resistance database (CARD) using Resistance Gene Identifier (RGI) with Perfect and Strict criteria requiring high-confidence matches. The high-quality/coverage sequence setting was used, corresponding to complete genomes, plasmids, or high-quality assemblies and excluding prediction of partial genes; Loose hits were excluded. The analysis suggested no evidence of acquired transferable antibiotic resistance genes. Although *B. adolescentis 150* harboured rifamycin-resistance genes, and most strains of *L. plantarum* exhibited genes resistant to vancomycin, these genes demonstrated low sequence identity and incomplete coverage and were also non-mobile. Similarly, screening against VFDB suggested no detectable virulence factors or pathogenic determinants in any strain.

### 2.3. Prediction of Adhesion and Mucin Interaction Potential

The adhesion potential of all 12 shortlisted psychobiotic strains was evaluated in silico based on predicted subcellular protein profiles. *L. plantarum NK151*, *UBLP40*, and *WLPL04* exhibited the greatest number of surface-associated proteins, extracellular/cell wall proteins, and predicted adhesins. Strains such as *L. plantarum PS128*, *L. acidophilus LA5*, *L. plantarum 90sk*, *L. rhamnosus UBLR58*, *L. helveticus IDCC3801*, *B. adolescentis 150*, and *B. breve UBBR01* exhibited a comparatively lower number of adhesion-associated proteins, indicating functional yet comparatively shorter mucosal retention. Due to the reduced number of surface-exposed proteins, the lowest adhesion score was observed for *L. helveticus R0052* and *B. breve M16V*.

### 2.4. Prebiotic-Psychobiotic Interaction Modeling

#### 2.4.1. Reconstruction and Curation of Genome-Scale Metabolic (GEMs) Network

NCBI database was used to retrieve the complete genomic sequences of candidate psychobiotic strains and to reconstruct the draft Genome-scale Metabolic models (GEMs) employing CarveMe; their corresponding protein FASTA files were used. Several inconsistencies, such as the presence of unbound reactions, incomplete metabolic pathways, and charge-to-mass stoichiometry imbalance, were initially found in the evaluation of these reconstructed GEMs. By temporarily turning off the nutrient uptake reactions and then carrying out the flux variability analysis, the unbound reactions in the model were identified [19]. Reactions exhibiting unrestricted flux ranges were considered infeasible or cycle-forming. Charge-to-mass stoichiometry imbalance was corrected by refining metabolite formulas and charges using reference databases such as PubChem, ChEBI, or BiGG. Proton imbalance was corrected by adding or removing protons within reaction equations [20]. The remaining gaps in the reaction were systematically resolved by gap-filling the curated GEMs using COBRApy, resulting in more physiologically consistent models in SBML format. The addition of these missing reactions restored the metabolic connectivity, decreased the pathway blockages, and thereby led to an enhancement in the integrity of the model [21,22]. The curated models encompassed not only central carbon metabolism but also amino acid biosynthesis pathways, cofactor cycles, and transport systems that were functional [19,23]. However, direct experimental validation of the reconstructed GEMs against published growth rates or substrate-utilization profiles was not performed because suitable strain-specific experimental datasets corresponding to the simulated conditions were not available. Consequently, the predicted growth and metabolic phenotypes should be interpreted as model-derived computational predictions, and experimental validation under defined conditions would be required to further establish the physiological relevance.

#### 2.4.2. Evaluation of Biomass Production and Flux Balance Analysis (FBA)

**Figure 2:**
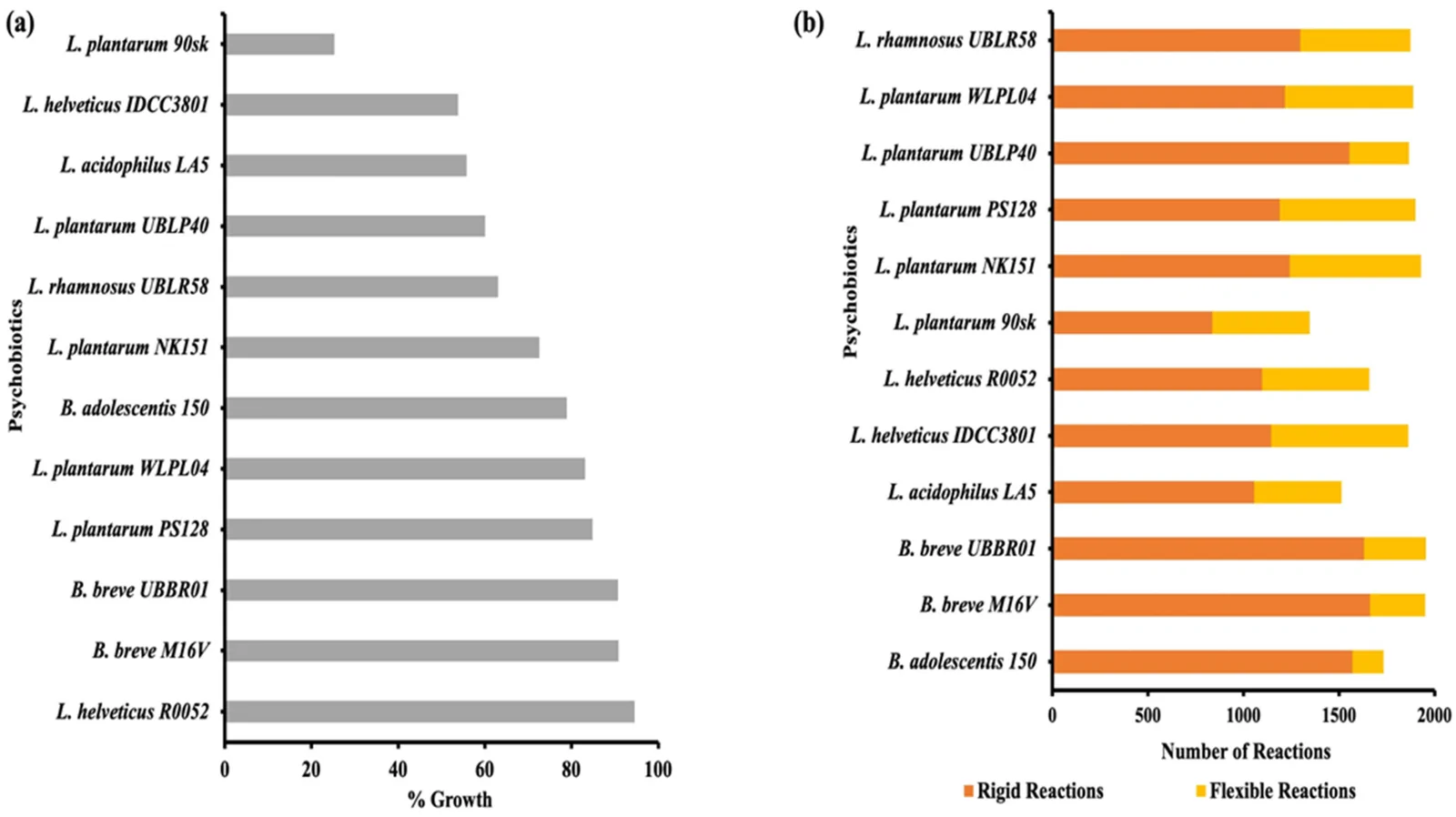
Psychobiotic strain selection. (**a**) Predicted biomass flux value (simulated % growth rate) of candidate psychobiotic strains under quercetin-supplemented conditions. (**b**) Distribution of rigid and flexible reactions across candidate psychobiotic strains obtained from flux variability analysis (FVA).

To investigate prebiotics and psychobiotic interactions, FBA was performed under quercetin-supplemented M9 basal medium. The predicted biomass flux values (simulated growth rate) demonstrated a substantial inter-strain metabolic heterogeneity [23]. Four strains, namely *L. helveticus R0052*, *B. breve M16V*, *B. breve UBBR01*, and *L. plantarum PS128*, exhibited the highest predicted biomass flux value, indicating the greater model-predicted metabolic capacity under quercetin-supplemented simulated conditions, as shown in Figure 2a. On the contrary, *L. plantarum 90sk* demonstrated the lowest predicted biomass flux value.

Analysis of nutrient uptake behavior, as shown in Table 2, predicted that ferrous ion (Fe^2+^), L-homoserine, nicotinamide mononucleotide, sulfate, and various sugars were preferentially consumed. In addition, uptake of oxygen in microaerophilic strains further differentiated strains with enhanced respiratory flexibility [23]. Secretion profile analysis predicted that the psychobiotic strains mainly produced acetate, CO_2_, ammonia, and organic acids. Acetate was secreted as a major metabolite by almost all strains, consistent with the typical fermentative behavior of *Lactobacilli* and *Bifidobacteria*.

#### 2.4.3. Metabolic Flexibility and Flux Variability Analysis (FVA)

To evaluate the metabolic robustness of each psychobiotic strain under the quercetin-supplemented growth conditions, flux variability analysis (FVA) was performed. Reactions with extremely narrow flux ranges (termed as rigid reactions) were predominant across most of the Genome-scale Metabolic models (GEMs). On the contrary, a subset of reactions displayed wider and more flexible flux ranges, reflecting metabolic adaptability. The number of flexible reactions varied significantly [Figure 2b], ranging from 163 in *B. adolescentis 150* to 552 in *L. rhamnosus UBLR58*.

Another key observation was the absence of unbounded reactions in all the Genome-scale Metabolic models (GEMs). Comparative strain analysis suggested that strains with higher predicted FBA biomass flux values exhibited a greater reaction flexibility. Strains such as *L. rhamnosus UBLR58*, *L. plantarum WLPL04*, and *B. breve UBBR01* demonstrated the highest flexibility. In contrast, strains like *L. plantarum 90sk* exhibited a smaller number of flexible reactions and a more constrained flux range. Based on the integrated evaluation of metabolic robustness, growth potential, and pathway flexibility, *B. breve UBBR01* and *L. plantarum PS128* were identified as promising psychobiotic strains for further investigations.

### 2.5. Network Pharmacology and Gut–Brain Axis Pathway Analysis

#### 2.5.1. Overview of Dataset Curation

A comprehensive three-layered dataset comprising 38 gut–brain metabolites, 76 host genes/proteins involved in neuroimmune and neurotransmission, and 46 quercetin -associated targets was systematically curated to characterize the molecular interactions along the gut–brain axis. Unlike the conventional network pharmacology approach that relies mainly upon computational interactions between compounds and diseases by utilising databases such as Similarity Ensemble Approach (SEA), BindingDB, GeneCards, DisGeNET, etc. [24,25]. The present work adopted a biologically driven, mechanism-oriented strategy highlighting the mechanistic pathways for quercetin-mediated modulation of the gut–brain axis.

#### 2.5.2. Selection of Gut–Brain Metabolites

A total of 38 gut microbially modulated neuroactive metabolites were identified based on the extensive literature survey. These metabolites were categorized into distinct functional groups as summarized in Table 3.

These metabolites act as key communication signals between gut microbes and the central nervous system and are responsible for the regulation of a variety of physiological processes, including vagal nerve activation, biosynthesis of neurotransmitters, immune–neural interaction, and modulation of the intestinal barrier [26,27,28].

#### 2.5.3. Selection of Host Genes and Proteins

This dataset consisted of 76 host genes and proteins involved in neurotransmission, synaptic plasticity, immune signaling, stress regulation, and redox balance. To ensure strong biological relevance to depression and the physiology of the gut–brain axis, these genes were collected from the KEGG database, Reactome, and a primary literature survey. The key mechanistic pathways included regulation of the hypothalamic–pituitary–adrenal (HPA) axis (CRH, CRHR1, NR3C1, FKBP5, etc.), GABAergic activity (GRIN1, GRM5, SLC1A2, GAD1, GAD2, etc.), monoamine signaling (MAO-A, MAO-B, COMT, DRD2, DRD3, SLC6A4, HTR1A, etc.), immune responses (TNF, IL1B, IL6, TLR4, etc.), oxidative stress (NOS, SOD, CAT, MPO, etc.), and neuroplasticity (BDNF, NTRK2, CREB1, MAPK, etc.) [29].

#### 2.5.4. Selection of Quercetin Targets

Using high-confidence interaction data from STITCH, STRING, Swiss Target Prediction, and ChEMBL, 46 quercetin-associated protein targets were identified. These targets exhibited strong alignment with the molecular pathways implicated in depression and gut–brain dysfunction. Key targets included monoamine-related proteins (MAO-A, MAO-B, CONT, SLC6A4, etc.), neuroprotective signaling proteins (AKT1, MAPK1, MAPK3, etc.), inflammatory and immune regulators (IL6, IL1B, TNF, NFKB1, PTGS2, etc.), and oxidative stress markers (SOD, NOS, NFE2L2, CAT, etc.) [30].

#### 2.5.5. Pathway Enrichment and Topology Analysis

Pathway Enrichment Analysis was performed using MetaboAnalyst 6.0 to elucidate the functional pathways associated with the curated set of gut-derived neuroactive metabolites.

Pathway Topology Analysis. Pathway topology analysis using the KEGG database for Homo sapiens identified several enriched pathways (*p* < 0.05, FDR corrected) as shown in Table 4. Among these, butanoate metabolism emerged as the most significantly enriched pathway (*p*-value = 5.76 × 10^−5^ and FDR value = 0.0046).

**Figure 3:**
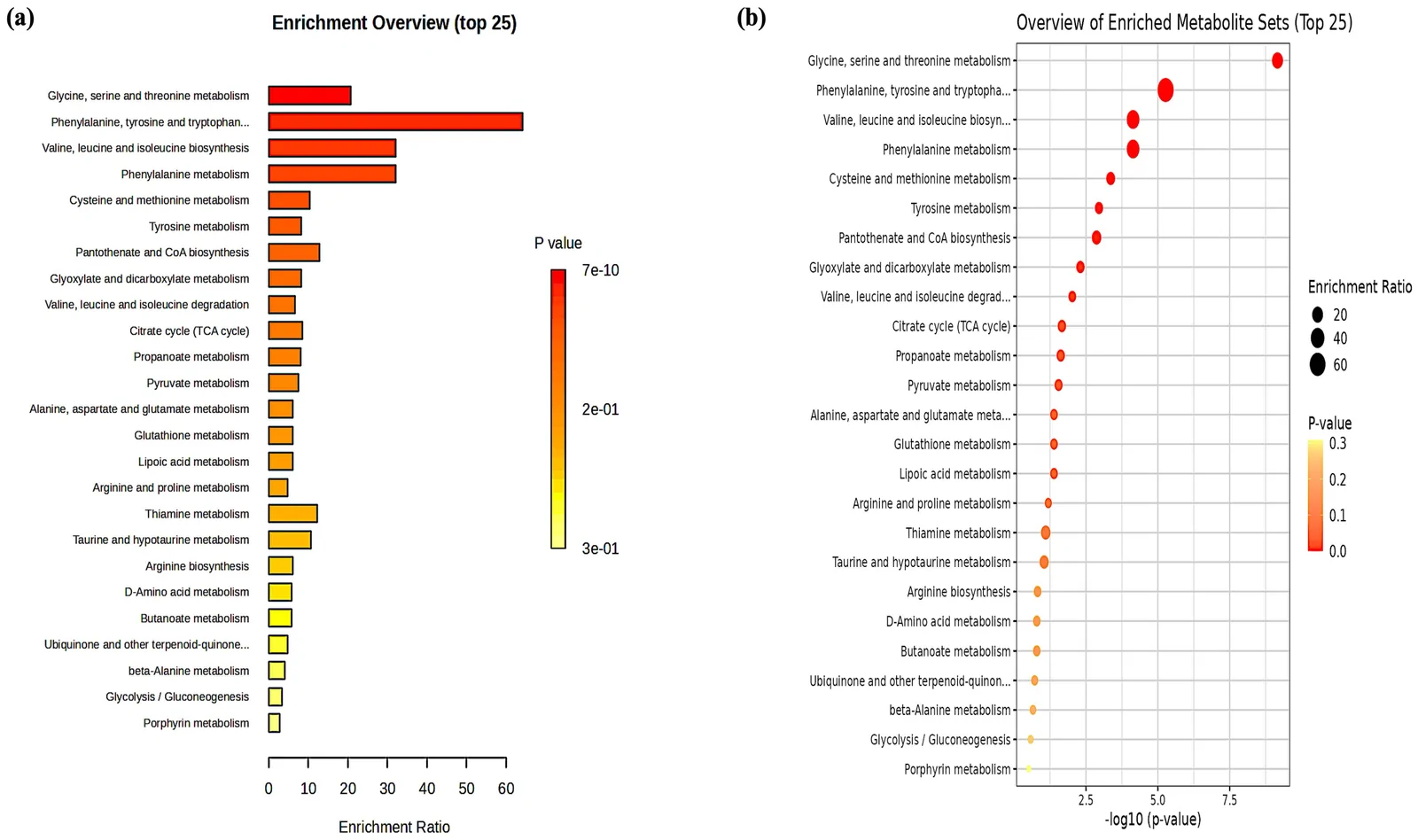
Metabolic Pathway enrichment analysis. (**a**) Bar plot showing the top 25 enriched metabolic pathways ranked based on enrichment ratio and statistical significance. (**b**) Bubble plot representing pathway enrichment significance, where the bubble size indicates enrichment ratio, and the color gradient reflects the statistical significance (*p*-value).

Pathway Enrichment Analysis. Pathway enrichment analysis suggested a predominance of amino acid-related pathways, as shown in Figure 3 and Table 5. Among these, glycine, serine, and threonine metabolism emerged as the most significant pathway (*p*-value = 6.85 × 10^−10^ and FDR value = 5.48 × 10^−8^).

Gene/Protein Pathway Analysis. Strong enrichment of amino acid-related pathways, with Metabolism of amino acids and derivatives identified as the most significant (*p* = 2.35 × 10^−6^; FDR = 1.12 × 10^−4^), was suggested by Reactome analysis. Table 6 depicts the enriched pathways associated with selected host genes involved in neurotransmission, metabolism, and immune signaling.

#### 2.5.6. Cytoscape Network Analysis

**Figure 4:**
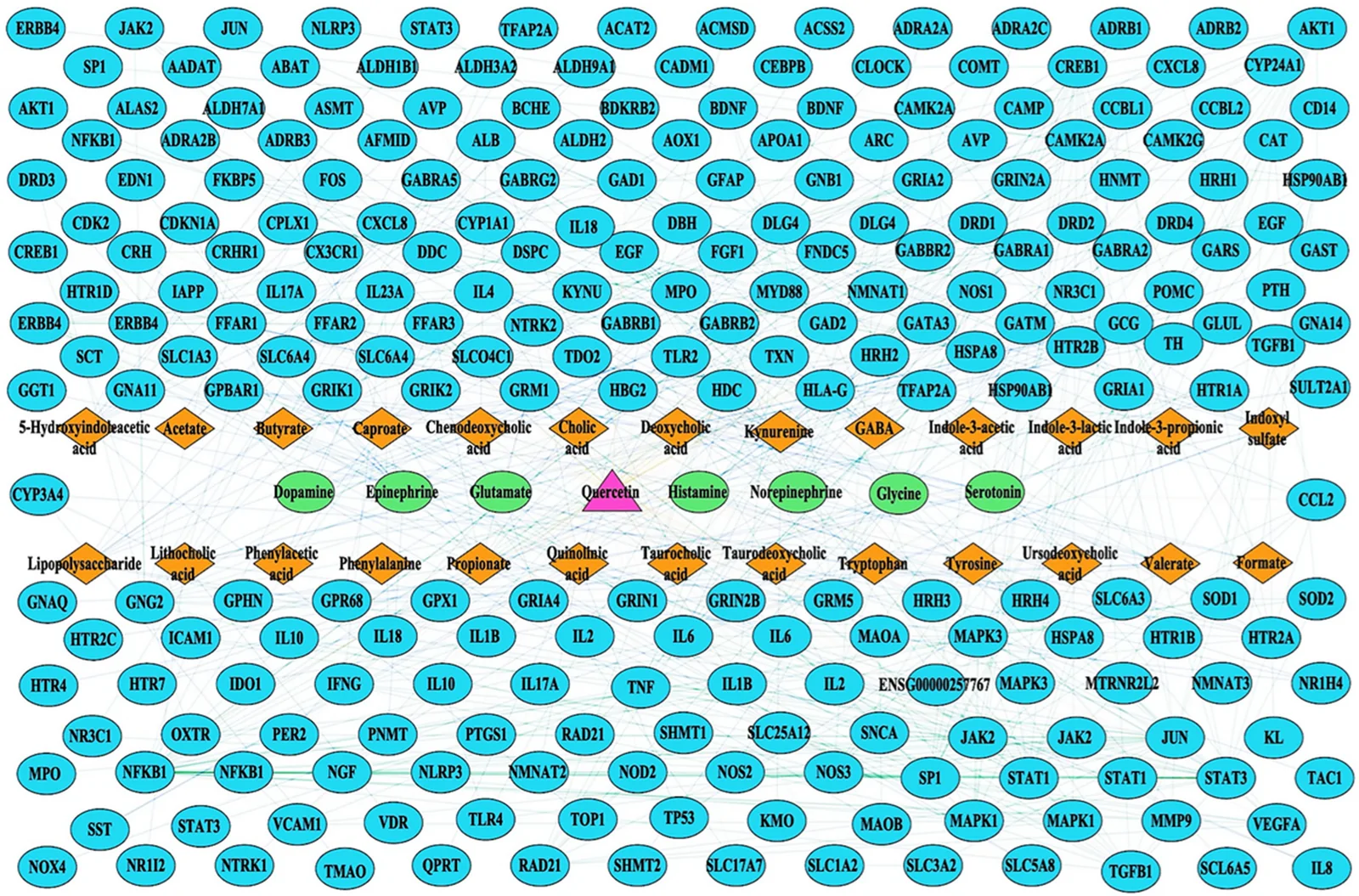
Integrated network pharmacology analysis network illustrating the interactions between quercetin, microbial metabolites, host genes, and signaling pathways. Blue circular nodes represent genes, orange diamond-shaped nodes represent metabolites, pink triangular nodes represent quercetin, and green circular nodes represent neurotransmitters, while edges indicate functional and regulatory associations.

Overview of Network and Identification of Hub Nodes. The curated metabolite-gene-quercetin dataset was imported into Cytoscape, and the network was constructed as shown in Figure 4. Within the network, blue circular nodes represent genes, orange diamond-shaped nodes represent metabolites, pink triangular nodes represent quercetin, and green circular nodes represent neurotransmitters. To identify the most influential genes within the network, topological analysis based on degree centrality using the Network Analyzer tool was performed. Quercetin showed the highest degree centrality (degree = 74). Several host genes, including NFKB1, JUN, STAT3, MAPK3, and STAT1, also showed high centrality values.

#### 2.5.7. Molecular Docking

**Figure 5:**
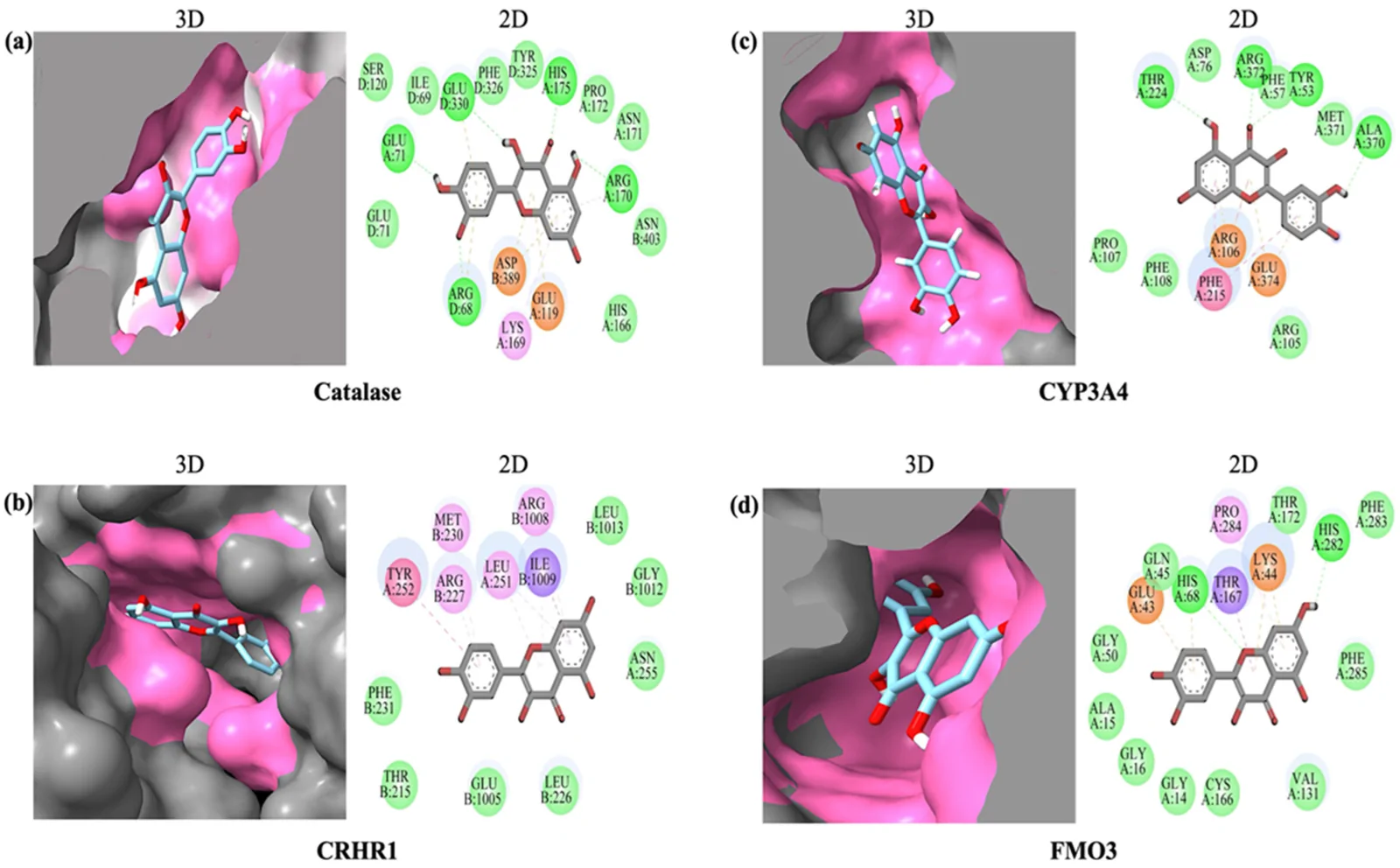
Molecular docking interaction analysis of quercetin with key target proteins (**a**) Catalase, (**b**) CRHR1, (**c**) CYP3A4, and (**d**) FMO3, having maximum binding affinity. The **left** panels represent the three-dimensional (3D) binding conformations of quercetin within the active binding pockets. In contrast, the **right** panels depict the corresponding two-dimensional (2D) interaction maps highlighting hydrogen bonding, hydrophobic interactions, π-π stacking, and electrostatic interactions with key amino acid residues.

**Figure 6:**
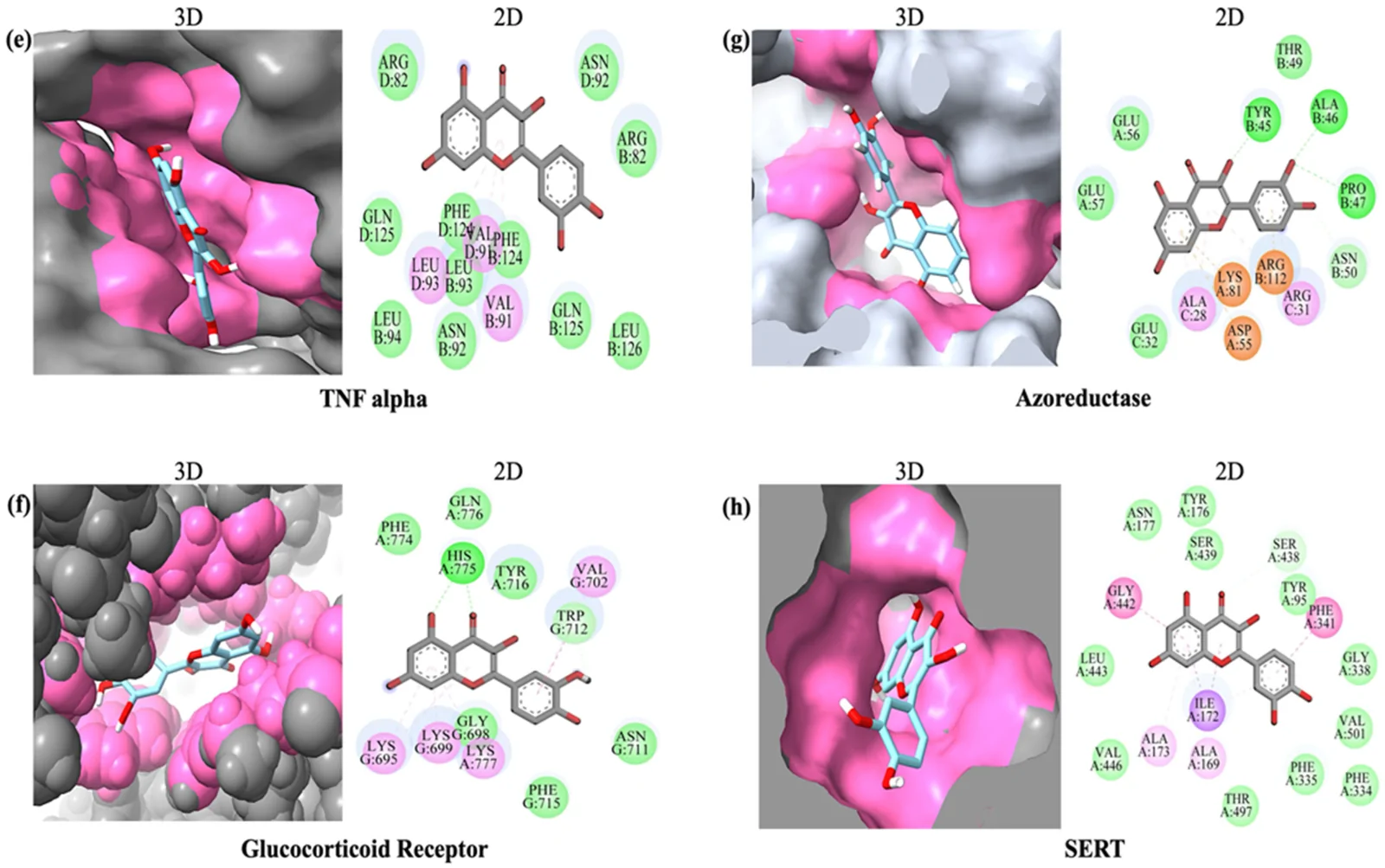
Molecular docking interaction analysis of quercetin with key target proteins (**e**) TNF-α, (**f**) Glucocorticoid receptor, (**g**) Azoreductase, and (**h**) SERT, having maximum binding affinity. The **left** panels represent the three-dimensional (3D) binding conformations of quercetin within the active binding pockets. In contrast, the **right** panels depict the corresponding two-dimensional (2D) interaction maps highlighting hydrogen bonding, hydrophobic interactions, π-π stacking, and electrostatic interactions with key amino acid residues.

To evaluate the binding affinity and interaction profile of quercetin with the selected protein targets, molecular docking was performed. The binding energies were in the range of −5.854 to −9.489 kcal/mol, suggesting a moderate to strong interaction potential with the functionally relevant active sites. The most favorable predicted binding affinity was observed with catalase (−9.489 kcal/mol), followed by CRHR1 (−8.932 kcal/mol), CYP3A4 (−8.848 kcal/mol), FMO3 (−8.819 kcal/mol), TNF-α (−8.754 kcal/mol), glucocorticoid receptor (−8.751 kcal/mol), SERT (−8.723 kcal/mol), azoreductase (−8.509 kcal/mol), and bile salt hydrolase (−8.295 kcal/mol) and, TLR4 (−8.004 kcal/mol) (Figure 5 and Figure 6). Moderately favorable predicted binding affinities were observed with COX-2 (−7.753 kcal/mol), IL-1β (−7.703 kcal/mol), MAO-B (−7.713 kcal/mol), TrkB (−7.899 kcal/mol), β-glucosidase (−7.860 kcal/mol), β-glucuronidase (3HN3: −7.533; 3LPF: −7.968 kcal/mol), GSK3β (−7.658 kcal/mol), CYP2C9 (−7.502 kcal/mol), CYP1A2 (−7.478 kcal/mol), PDE4 (−7.276 kcal/mol), quercetinase (−7.067 kcal.mol), and ERK2 MAPK (−7.007 kcal/mol). Comparatively lower predicted binding affinities were observed for Akt/PKB (−5.854 kcal/mol), SOD1 (−6.315 kcal/mol), PKC-δ (−6.322 kcal/mol), and MAO-A (−6.831 kcal/mol) [31]. The binding was predominantly mediated through conventional hydrogen bonds involving key polar and charged residues (such as Glu, Asp, Arg, Lys, His, Asn, Gln, Ser, and Thr), enabling strong electrostatic stabilization within catalytic and regulatory pockets. 2D and 3D structures of the remaining protein targets are provided in Supplementary Figures S1–S5.

#### 2.5.8. Redocking Validation

To evaluate the reproducibility of the docking protocol, the crystallographic reference ligand was redocked into its corresponding binding site, and the predicted poses were compared with the ligand coordinates of the experimentally determined crystallographic pose using RMSD. It was observed that all the poses were successfully reproduced with an RMSD value of less than 2.0 Å, indicating satisfactory reproduction of the experimentally observed binding orientations [32,33].

#### 2.5.9. Molecular Dynamics (MD) Simulation

**Figure 7:**
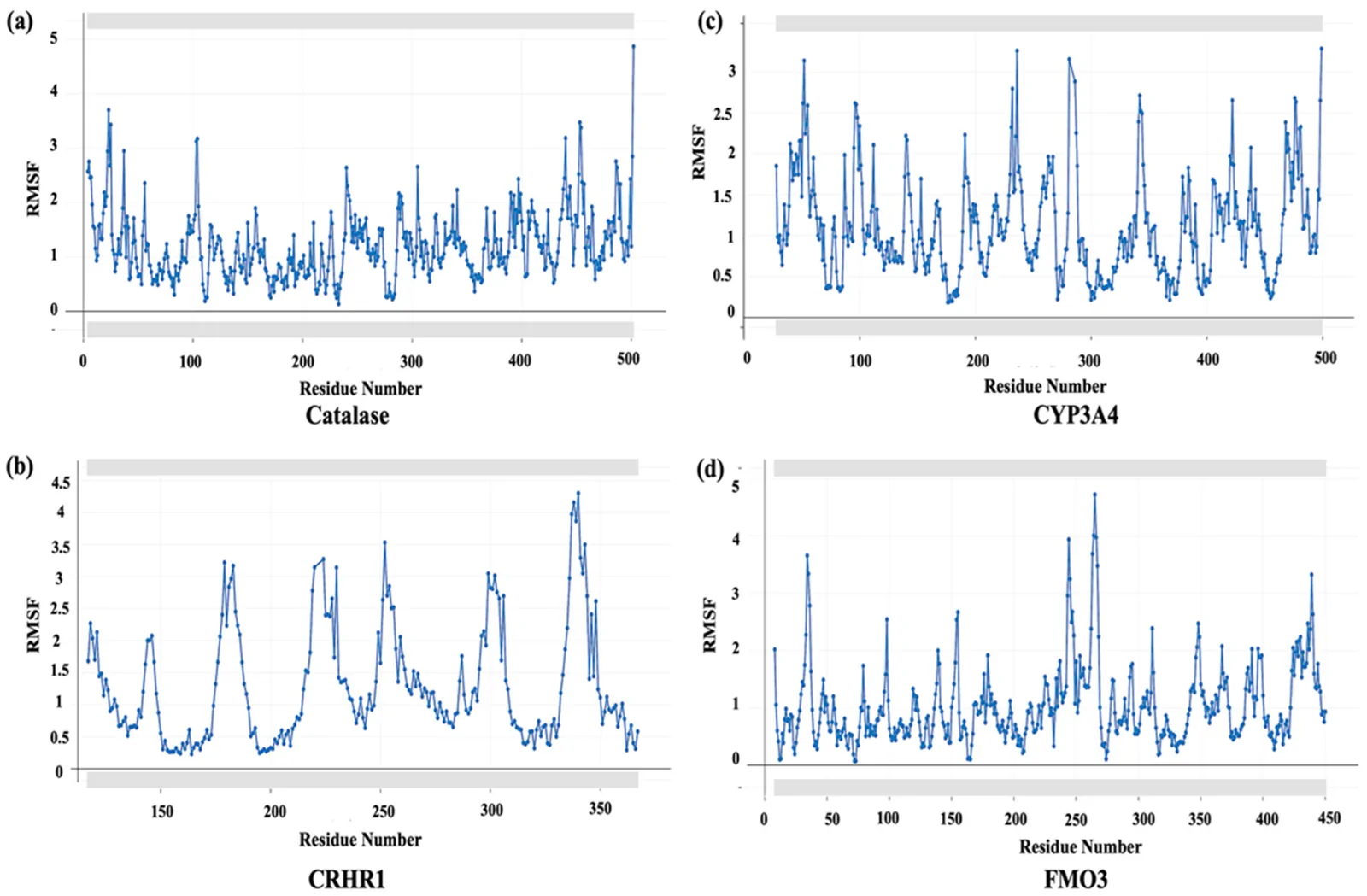
Root mean square fluctuation (RMSF) plots depicting structural stability and residue-level flexibility of protein–quercetin complexes during molecular dynamics simulation for (**a**) Catalase, (**b**) CRHR1, (**c**) CYP3A4, and (**d**) FMO3.

**Figure 8:**
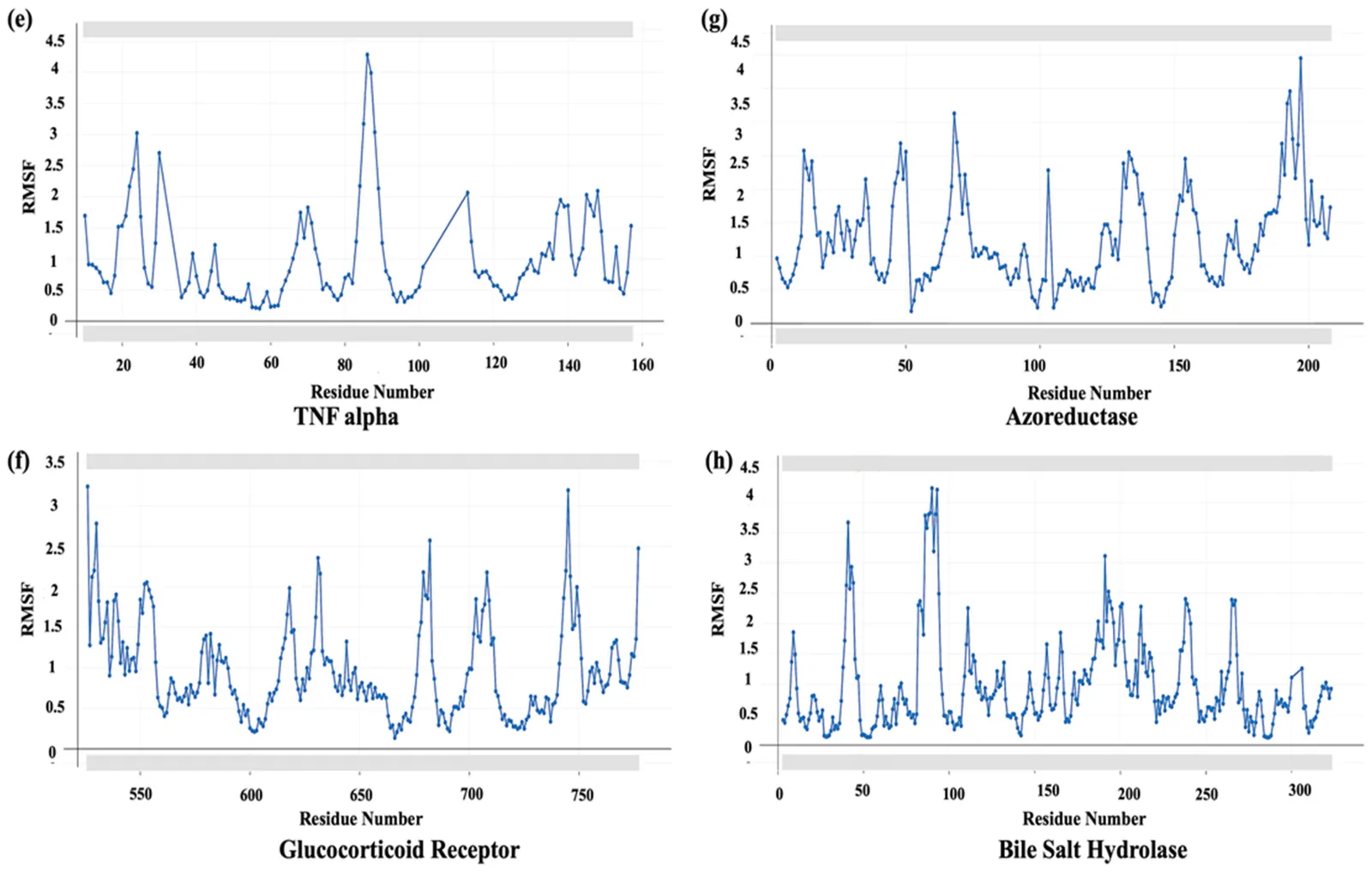
Root mean square fluctuation (RMSF) plots depicting structural stability and residue-level flexibility of protein–quercetin complexes during molecular dynamics simulation for (**e**) TNF-α, (**f**) Glucocorticoid receptor, (**g**) Azoreductase, and (**h**) Bile salt hydrolase.

Molecular 3.0 to evaluate the dynamic stability and structural integrity of quercetin-bound protein targets associated with the gut–brain axis. Both root mean square fluctuation (RMSF) and root mean square deviation (RMSD) analyses were used to evaluate the structural stability. The mean RMSF value ranged from 0.909 Å (PKC-δ) to 1.645 Å (Akt/PKB), with more than 75% of all proteins showing average fluctuations within a narrow range of 1.0–1.4 Å. Figure 7 and Figure 8 illustrate the RMSF profiles of quercetin-bound protein complexes with the highest binding affinities. Notably, CYP3A4 (1.097 Å), quercetinase (1.081 Å), glucocorticoid receptor (1.047 Å), PDE4 (1.050 Å), PKC-δ (0.909 Å), and TLR4/MD2 (0.963 Å) demonstrated low average RMSF values, suggesting that the ligand-bound state has a very compact structural state. Proteins such as Akt/PKB (1.645 Å), TrkB (1.440 Å), ERK2 (1.406 Å), SOD1 (1.397 Å), and MAO-A (1.351 Å) exhibited moderate fluctuations in their flexibility [34]. Some of the individual trajectories exhibited occasional isolated peak fluctuations of approximately 5–10 Å; these fluctuations were all limited to either terminal segments or solvent-exposed loop regions of the proteins. Critically, the catalytic domains and primary ligand-binding domains of these proteins showed no fluctuations during the simulations. Furthermore, there was no indication of destabilization or conformational collapse of the protein structure as a result of the applied simulation conditions.

RMSD analysis across 1000 simulation frames showed mean backbone RMSD ranging from 3.347 Å (TLR4/MD2) to 5.399 Å (Akt/PKB). The majority of proteins exhibited a mean RMSD value ranging from 3.3 to 4.6 Å. Proteins such as TLR4/MD2, TNF-α, CYP3A4, β-glucuronidase, quercetinase, PDE4, FMO3, glucocorticoid receptor, and catalase exhibited lower RMSD values (<3.8 Å). Proteins such as GSK3β, ERK2, SERT, SOD1, PKC-δ, COX-2, CYP isoforms, CRHR1, and TrkB exhibited moderate RMSD values. Although a few proteins (Akt/PKB, MAO-A, and Azoreductase) had relatively higher mean RMSD values (>5.0–5.4 Å), no evidence of continued progressive structural drift, abrupt oscillations in the trajectory, or any significant large-scale unfolding events was observed. Additionally, low standard deviation indicated stable and consistent trajectory behaviour.

Contact map analysis further suggested the integrity of the tertiary structure and also the intrachain interactions, with no evidence of significant unfolding or conformational collapse in any of the protein systems studied. Furthermore, throughout the course of the simulations, the α-helices and β-sheets were structurally intact.

#### 2.5.10. ADMET Profiling

**Figure 9:**
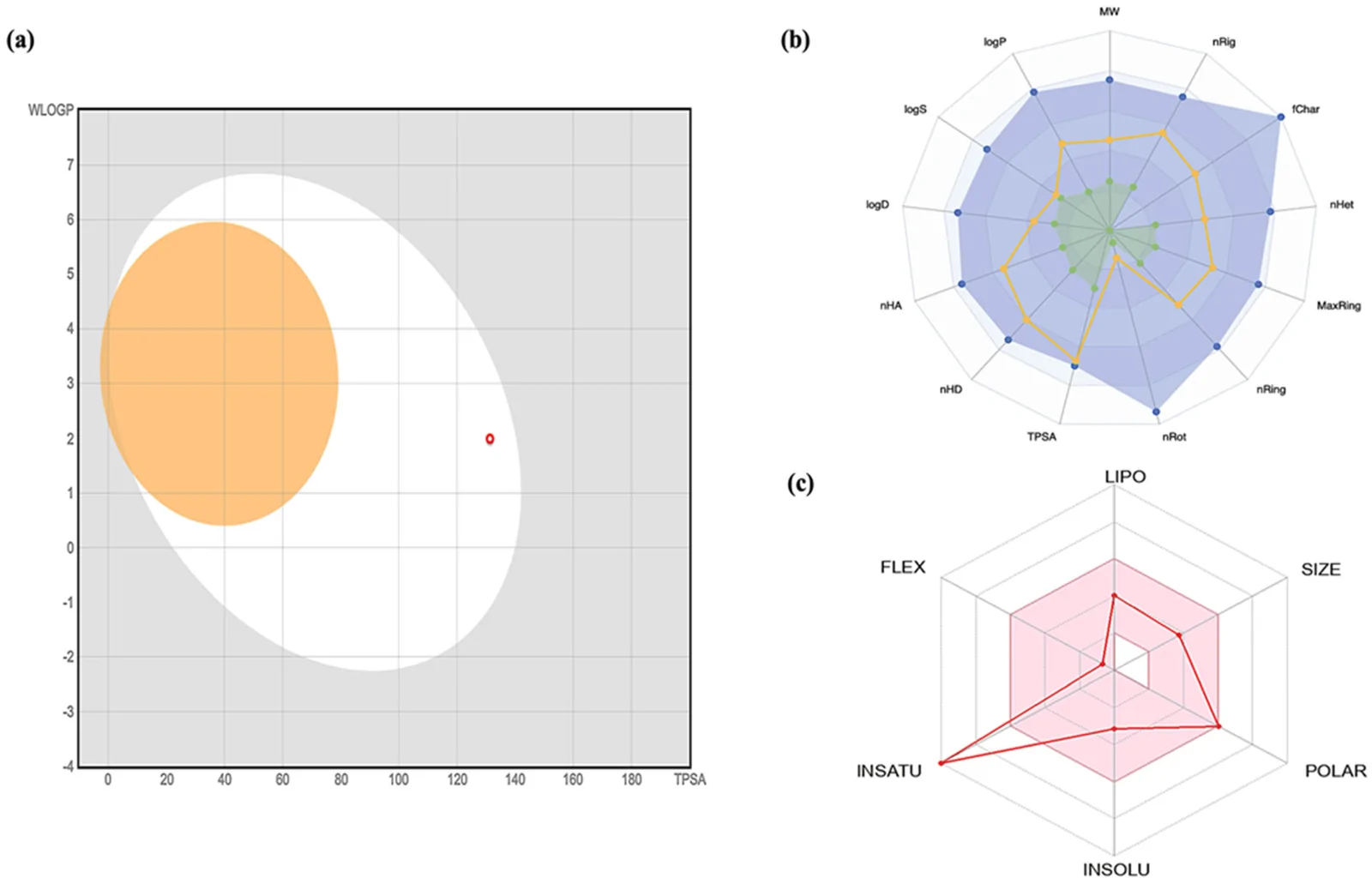
In silico ADME and drug-likeness evaluation of quercetin. (**a**) Boiled-Egg model predicting gastrointestinal absorption and blood–brain barrier permeability of quercetin based on lipophilicity and topological polar surface area (TPSA). (**b**) Physicochemical Radar plot representing major pharmacokinetic parameters of quercetin. (**c**) Bioavailability radar depicting the various properties of quercetin.

A favorable pharmacokinetic and safety profile of quercetin was predicted by the results of ADMET profiling. Quercetin demonstrated good intestinal absorption (with a predicted value of ~77%). It has a moderate CaCO-2 permeability and is classified as a P-glycoprotein substrate. Pharmacokinetic distribution analysis indicated moderate systemic distribution (VDss 1.559 log L/kg) with partial plasma protein binding (fraction unbound 0.206). Low predicted blood–brain barrier permeability (−1.098 log BB) and low CNS permeability (−3.065 log PS). This finding was further corroborated by the boiled-egg model as shown in Figure 9a.

The value of predicted total clearance was 0.407 log ml/min/kg, with no involvement of renal OCT2 transporter. Favorable toxicity predictions were observed, with no predicted mutagenicity, hepatotoxicity, or hERG-related cardiotoxicity risks. Evaluation by SwissADME also indicated that quercetin satisfies major drug-likeness criteria, including Lipinski, Ghose, Veber, Egan, and Muegge filters, without any violations [35]. The physicochemical radar [Figure 9b] demonstrated that key properties such as molecular weight, lipophilicity, polarity, and flexibility fall within optimal ranges for oral bioavailability. Additionally, the bioavailability radar [Figure 9c] indicated a balanced profile, with acceptable lipophilicity, size, polarity, and solubility parameters. Quercetin exhibited a molecular weight of 302 g/mol and moderate lipophilicity, with a consensus LogP ≈ 1.2–2.0. Its topological polar surface area (131 Å^2^), together with five hydrogen bond donors and seven hydrogen bond acceptors, reflected a balanced polarity profile compatible with oral administration. Additionally, the predicted bioavailability score of 0.55 supports its suitability as an orally active compound.

## 3. Discussion

The present study employed an integrated in silico systems biology approach combining comparative genomics, genome-scale metabolic modelling, network pharmacology, molecular docking, molecular dynamics and ADMET profiling to investigate the potential therapeutic relevance and molecular interactions of a quercetin–psychobiotic co-delivery system for the treatment of depression via modulation of the gut–brain axis. The genomic sequences of all 12 candidate psychobiotic strains were successfully retrieved from the NCBI Genome Database in both GenBank and FASTA formats, indicating genomic complexity and metabolic versatility. Subsystem-level categorization of the candidate psychobiotic strains was obtained using RAST-based Functional Genome Annotation. The results suggested the presence of genes involved in the production of neuroactive metabolites across all candidate strains. The presence of genes involved in the production of glutamate, particularly the glutamate decarboxylase system, suggested a strong potential for the synthesis of gamma-aminobutyric acid (GABA). Also, the presence of genes encoding GABA transporters and the absence of the genes associated with the degradation of GABA suggested their ability to accumulate GABA rather than its degradation, contributing to enhanced immunomodulatory effects [36]. Similar results have been reported by Bai et al. for *Lactobacillus plantarum* SY1, which is involved in the production of GABA and also exhibited antioxidant and neuromodulatory activity, supporting the significance of the glutamate decarboxylase system in the selection of psychobiotics [36]. Whereas the presence of the genes involved in the metabolism of tryptophan indicated a contribution to serotonin-mediated pathways [26,37]. More recently, Qian et al., 2024, reported the antidepressant activity of the strains of *Bifidobacterium breve*, which were capable of producing indole-3-lactic acid (ILA), indicating the importance of the Aldh pathway in the production of ILA [10]. The results unveiling the presence of core genes involved in the production of acetate and the absence of the classical butyrate-producing genes across all strains are consistent with established scientific evidence showing that *Lactobacillus* and *Bifidobacterium* are not the primary producers of butyrate. Through the production of acetate and involvement in cross-feeding interactions within the gut microbiota, they indirectly contribute to the production of butyrate [27,38]. These results were in corroboration with the findings of Kim et al., 2025 [39]. In their study, stool metagenomes of 133 patients with depression and 532 individuals without depression were analysed, and it was observed that SCFA production activity decreased in the patients suffering from depression due to depletion of biosynthetic pathways involved in SCFA production. Various genes associated with the interaction of the mucosal surface, modulation of the immune system, and gut barrier function were also found across all strains. The presence of these genes indicates a strong potential for promoting stronger and more stable colonisation. Furthermore, the presence of the bile salt hydrolase in the majority of strains supported their role in host lipid and immune modulation [3,40]. Moreover, the presence of genes associated with oxidative stress tolerance and redox-balancing suggested enhanced survival and functional ability of these strains under GIT conditions owing to the presence of an antioxidant and redox-balancing system [41]. In addition, genes involved in the biosynthesis of vitamins and cofactors were detected across all the strains, particularly vitamin B6, which plays an important role in neurotransmitter synthesis and neurochemical regulation, thereby contributing to the psychobiotic potential of these strains [42].

For the evaluation of genomic safety, all the strains were screened against the comprehensive antibiotic resistance database. The analysis predicted no evidence of acquired transferable antibiotic resistance genes, and the absence of detectable virulence factors or pathogenic determinants in any strain [43]. The adhesion potential of all 12 shortlisted psychobiotic strains was evaluated in silico based on predicted subcellular protein profiles. Strains of *L. plantarum* exhibited the greatest number of surface-associated proteins, extracellular/cell wall proteins, and predicted adhesins. These features suggest a strong intrinsic capacity of these strains for mucin binding and intestinal colonization. Strains of *Bifidobacterium* exhibited a comparatively lower number of adhesion-associated proteins, indicating functional yet comparatively shorter mucosal retention. Therefore, as strongly adherent *Lactobacilli* may enhance mucosal contact, and metabolically active *Bifidobacteria* may contribute to the complementary biochemical functions in the gut–brain axis, a synergistic psychobiotic formulation strategy may be supported by these results.

To investigate prebiotics and psychobiotic interactions, FBA was performed under quercetin-supplemented M9 basal medium. This approach was used to predict the inter-strain variability in biomass fluxes (growth rates), nutrient uptake behavior, and secretion profile of candidate psychobiotic strains under quercetin-supplemented M9 basal medium. Four strains, namely *L. helveticus R0052*, *B. breve M16V*, *B. breve UBBR01*, and *L. plantarum PS128*, exhibited the highest predicted metabolic flux value, suggesting a greater metabolic adaptability. On the contrary, *L. plantarum 90sk* demonstrated the lowest biomass flux, which could be attributed to the limited metabolic flexibility [19]. Analysis of nutrient uptake behavior highlighted the importance of amino acid precursor acquisition and redox balancing. Secretion profile analysis suggested that the psychobiotic strains mainly produced acetate, CO_2_, ammonia, and organic acids. These observations corroborated literature recognizing acetate as a central metabolite and a major contributor in the microbial cross-feeding network [44]. The increased levels of acetate production further supported the neuroactive potential of these candidate psychobiotic strains, given the established roles of short-chain fatty acids in regulating host metabolism and modulating the gut–brain axis [27,28,41].

To evaluate the metabolic robustness of each psychobiotic strain under the quercetin-supplemented growth conditions, flux variability analysis (FVA) was performed. The predominance of reactions with extremely narrow flux ranges across most of the GEMs indicated the presence of highly constrained core metabolic pathways. This finding aligns with the results of previous studies where essential pathways such as central carbon metabolism, amino acid and nucleotide synthesis, and energy production function within very tight flux limits [19]. These pathways are vital for sustaining growth and therefore exhibit minimal flexibility under optimal conditions. On the contrary, the number of flexible reactions varied significantly, suggesting species-specific metabolic adaptability [23]. Most of these flexible reactions are associated with carbohydrate breakdown, redox balancing, short-chain fatty acid synthesis, and transport activities. Similar trends have been reported in microbial systems, where auxiliary catabolic and fermentation pathways exhibited great flux variability, enabling adaptive responses to environmental changes [19]. Another key observation was the absence of unbounded reactions in all the Genome-scale Metabolic models (GEMs). This indicated the robustness of the curated model due to the successful removal of thermodynamically infeasible cycles (TICs) during model refinement and also validated the efficiency of gap filling and directional corrections [22]. Comparative strain analysis predicted that strains with higher predicted FBA biomass flux values exhibited a greater reaction flexibility. Strains such as *L. rhamnosus UBLR58*, *L. plantarum WLPL04*, and *B. breve UBBR01* demonstrated the highest flexibility, suggesting that they can redirect carbon and energy through different pathways without compromising growth. This adaptability is particularly advantageous in the presence of quercetin, where aromatic metabolism and NAD^+^-linked reactions may need dynamic rearrangement. In contrast, strains like *L. plantarum 90sk* exhibited a smaller number of flexible reactions and a more constrained flux range, suggesting a lower metabolic redundancy and a limited adaptability to changing nutrient environments [23]. Tarzi et al. highlighted similar advantages of genome-scale metabolic modelling for resolving strain- and community-level metabolic differences that cannot be inferred from taxonomic classification alone. The metabolic modelling can provide mechanistic insight into host–microbe interactions by linking microbial metabolic capacity with metabolite production [45].

Pathway Enrichment Analysis was performed to elucidate the functional pathways associated with the curated set of gut-derived neuroactive metabolites. Results of Pathway topology analysis suggested that butanoate metabolism emerged as the most significantly enriched pathway, driven largely by the presence of butyrate, acetate, and other related SCFAs. These metabolites are well recognized for their anti-inflammatory and neuroprotective roles [27,28,41]. Dalile et al. described SCFA-mediated signaling as an important route linking microbial metabolism with neural and endocrine processes [27]. Pathway enrichment analysis indicated a predominance of glycine, serine, and threonine metabolism pathways, which are major contributors to NMDA receptor regulation, inhibitory neurotransmission, and neurochemical homeostasis [41]. Reactome analysis showed the strong enrichment of amino acid-related pathways, with metabolism of amino acids and derivatives identified as the most significant pathway. The genes involved in the synthesis, degradation, and conversion of key neurotransmitter precursors were included in this pathway, showing its importance in the maintenance of monoamine balance and overall neuronal functioning [29]. Tian et al. linked the antidepressant effects of *B. breve* CCFM1025 with modulation of gut microbial tryptophan metabolism [6]. The curated metabolite-gene-quercetin dataset was imported into Cytoscape, and the network was constructed. To identify the most influential genes within the network, topological analysis based on degree centrality was performed. Quercetin showed the highest centrality value, indicating extensive interaction with genes involved in inflammatory signaling, neurotransmitter regulation, and cellular stress responses. Several host genes, including NFKB1, JUN, STAT3, MAPK3, and STAT1, also showed the highest centrality value, suggesting their major role in modulating neuroinflammatory and signaling pathways along the gut–brain axis [29,30]. Debnath et al. also reported quercetin as a multifunctional compound capable of influencing inflammatory, oxidative, neurotrophic, and intracellular signaling pathways [46].

To evaluate the binding affinity and interaction profile of quercetin with the selected protein targets, molecular docking was performed. The most favorable predicted binding affinity was observed with catalase, followed by CRHR1, CYP3A4, FMO3, TNF-α, glucocorticoid receptor, azoreductase, and bile salt hydrolase. These results suggest a pronounced affinity of quercetin towards antioxidant enzymes, stress-axis regulators, inflammatory mediators, microbial enzymes, and hepatic metabolic proteins. Moderately favorable predicted binding affinities were observed with COX-2, IL-1β, MAO-B, TrkB, β-glucosidase, β-glucuronidase, TLR4, and GSK3β. These interactions indicate potential modulation of inflammatory cascades, monoaminergic pathways, neurotrophic signaling, and gut microbial enzymatic processes. The binding was predominantly mediated through conventional hydrogen bonds involving key polar and charged residues. The multiple hydroxyl groups of quercetin played a central role in hydrogen bond formation, while the planar aromatic scaffold facilitated extensive π–π stacking, π–alkyl, and π–sigma interactions with hydrophobic residues, including Phe, Tyr, Trp, Leu, Ile, Val, Ala, and Pro. This interaction pattern across diverse protein classes highlights that quercetin possesses a structurally adaptable binding mode, enabling simultaneous modulation of oxidative stress, neuroinflammation, stress signaling, microbial metabolism, and neurotransmitter regulation [30].

Results of redocking validation also suggested that all the poses were successfully reproduced with an RMSD value of less than 2.0 Å, indicating satisfactory reproduction of the experimentally observed binding orientations [32,33].

Molecular dynamics simulations were performed to evaluate the dynamic stability and structural integrity of quercetin-bound protein targets associated with the gut–brain axis. The RMSF analysis showed variations in the residue-level flexibility, and RMSD analysis further indicated the extent of conformational deviation within each simulation. The majority of proteins exhibited a mean RMSD value ranging from 3.3 to 4.6 Å [34]. However, these values were interpreted for each of the individual protein systems rather than as a direct measure for ranking the stability of different proteins because of the differences in size, structures, and conformational flexibility of proteins. Hollingsworth and Dror also emphasized that molecular-dynamics-derived RMSD and RMSF provide information on conformational behavior rather than direct measures of biological activity [34]. Contact map analysis further suggested the integrity of the tertiary structure and also the intrachain interactions, with no evidence of significant unfolding or conformational collapse in any of the protein systems studied. Overall, the CABS-flex simulations suggested that the predicted quercetin-bound complexes maintained their overall structural characteristics during the simulated conformational sampling [30].

Oral administration of quercetin was demonstrated by its good intestinal absorption. However, moderate CaCO-2 permeability and classification as a P-glycoprotein substrate suggested the potential transporter-mediated efflux of quercetin at the intestinal barrier. While this may limit systemic bioavailability, it could enhance luminal exposure and interaction with gut microbiota [35,47]. Low predicted blood–brain barrier permeability and low CNS permeability indicated minimal direct CNS penetration, supporting the predominant involvement of peripheral mechanisms, including modulation of inflammatory and gut-derived signaling pathways [26,48]. Li et al. reported that quercetin attenuated CUMS-induced depression-like behaviors while simultaneously reshaping gut microbiota and altering brain metabolic profiles [49]. Notably, docking and MD analyses showed stable binding to CYP isoforms and gut microbial enzymes, suggesting that quercetin may participate in both host- and microbiota-mediated metabolic modulation. The predicted total clearance was moderate, with no involvement of the renal OCT2 transporter, suggesting predictable elimination kinetics.

## 4. Methodology

### 4.1. Psychobiotic Strain Selection

#### 4.1.1. Information Sources and Selection Procedure

A comprehensive and systematic literature survey was conducted using major scientific databases, including PubMed, Web of Science, and Scopus, to identify candidate psychobiotic bacterial strains. Various keywords such as “psychobiotics”, “gut-brain axis”, “neuroactive metabolites”, “*Bifidobacterium*”, “*Lactobacillus*”, and “depression” were used for searching the databases. Initially, 41 candidate strains belonging to the genera *Bifidobacterium* and *Lactobacillus* were selected based on specific inclusion criteria. These criteria included: (a) reported psychobiotic activity or neuroactive potential, (b) documented evidence of participation in pathways associated with the production, metabolism, or regulation of neurotransmitters, and (c) reported significance to gut–brain communication [43,44,50]. Now, as complete genomic information was required for comparative genomic and genome-scale metabolic modelling, another major additional factor in the selection was the availability of a complete genomic sequence in public repositories. Therefore, after screening for genomic availability, strains for which genomic information was not available in public repositories were excluded. Consequently, 12 psychobiotic bacterial strains satisfying the inclusion criteria and genomic data availability criteria were finalized for subsequent computational analyses.

#### 4.1.2. Retrieval of Whole Genome Sequence

The complete genomic sequences of 12 shortlisted psychobiotic strains were retrieved from the National Center for Biotechnology Information (NCBI) Genome database (https://www.ncbi.nlm.nih.gov/datasets/genome/) (accessed on 25 June 2025) in GenBank (.gbk) or FASTA (.fna/.faa) format. Each genomic dataset was examined for completeness and formatted consistently prior to analysis so as to ensure reliable functional and metabolic profiling [51,52].

#### 4.1.3. Functional Genome Annotation

The whole bacterial genomes of selected strains were functionally annotated using the Rapid Annotation using Subsystem Technology (RAST) server (https://rast.nmpdr.org/rast.cgi) (accessed on 12 July 2025) [53]. The Comprehensive Antibiotic Resistance Database (CARD, version 4.0.1) (https://card.mcmaster.ca/analyze/rgi) (accessed on 27 July 2025) was employed to identify antibiotic-resistant genes, while the Virulence Factors Database (VFDB) (https://www.mgc.ac.cn/cgi-bin/VFs/v5/main.cgi) (accessed on 11 August 2025) was used to screen for the presence of virulence-associated genes [53,54].

#### 4.1.4. Prediction of Adhesion and Mucin Interaction Potential

The adhesion potential of all 12 shortlisted psychobiotic strains was evaluated in silico to assess their adhesion potential with intestinal mucosa. RAST annotation and Prodigal (RAST toolkit) were used to predict the open reading frames (ORFs) and coding protein sequences (CDS) from each genome, generating protein FASTA (.faa) files. The resulting protein sequences were analyzed for subcellular localization using the pSORTb Subcellular Localization Prediction Tool (https://www.psort.org/psortb/) (version 3.0.3) (accessed on 3 October 2025). Proteins localized to the cell wall, cytoplasmic membrane, and extracellular space were shortlisted as potential adhesin-related candidates [55,56].

### 4.2. Prebiotic-Psychobiotic Interaction Modeling

#### 4.2.1. Reconstruction of Psychobiotic Genome-Scale Metabolic (GEMs) Network

The complete genomic sequences of 12 shortlisted psychobiotic strains were retrieved from the NCBI database. Protein sequence data (FASTA format) of each strain were utilized to reconstruct the draft Genome-scale Metabolic models (GEMs) employing CarveMe (version 1.6.5). The initial draft reconstructed models contained incomplete and unbound reactions along with charge and mass stoichiometric imbalances. These deficiencies were addressed through gap-filling procedures to improve network completeness and physiological relevance. Gap filling was performed using the CarveMe optimization-based framework, in which candidate reactions from the universal reaction network are evaluated to restore metabolic functionality while minimizing the penalty associated with reaction addition. Reactions supported by genomic annotation evidence are preferentially selected during this process. The gap-filling procedure was formulated as an optimization problem in which the minimum set of candidate reactions required to satisfy the metabolic feasibility criterion was selected. The default CarveMe parameters were retained, including a minimum biomass-production constraint of 0.1 mmol gDW^−1^ h^−1^, a maximum reaction flux (bigM) of 1000 mmol gDW^−1^ h^−1^, and an activity tolerance of 10^−9^. The draft models were curated using COBRApy version 0.24.0 in Python in SBML format [51,57]. The model characteristics, composition of M9 media, biomass objective information, and GEM reconstruction and gap-filling parameters are provided in Supplementary Tables S1–S4. Also, the complete stoichiometric equation of the Growth reaction is provided in Supplementary Table S3.

#### 4.2.2. Flux Balance Analysis and Growth Prediction

A constraint-based reconstruction and analysis (COBRA) framework was used to simulate the metabolic behaviour of candidate psychobiotic strains under varying nutrient conditions. The Flux Balance Analysis (FBA) was applied as it effectively captures steady-state metabolic activity and predicts flux distributions across large biochemical networks [45].

Each genome-scale metabolic model (GEM) was mathematically represented by a stoichiometric matrix (S) of dimensions *m* × *n*, where *m* denotes the number of metabolites and *n* represents the number of biochemical reactions in the network. The steady-state assumption, where intracellular metabolite concentrations remain constant over time, was expressed as:

(1) S⋅v=0

where *v* is an *n* × 1 flux vector representing the rate of each metabolic reaction.

In this matrix, the element *S_ij_* denotes the stoichiometric coefficient of metabolite *i* in reaction *j*: negative for reactants, positive for products, and zero if the metabolite is not involved. This ensures mass conservation for all metabolites in metabolic network. To simulate the defined metabolic conditions, reaction-specific lower and upper flux bounds encoded in the reconstructed SBML models were retained. Reversible reactions were allowed to carry bidirectional flux, whereas irreversible reactions were constrained to non-negative flux. The default bounds for reversible reactions were −1000 to 1000 mmol gDW^−1^ h^−1^, whereas irreversible reactions were constrained between 0 and 1000 mmol gDW^−1^ h^−1^ and reverse-only reactions between −1000 and 0 mmol gDW^−1^ h^−1^

(2) $$v_{min,j}\leq v_{j}\leq v_{max,j}$$

Irreversible reactions were constrained such that *v_min_*_,*j*_ = 0, while reversible reactions were allowed bidirectional flux (*v_min_*_,*j*_ < 0). Nutrient exchange and transport reactions were adjusted according to the composition of the simulated media. The objective function was defined as the maximization of biomass formation, representing the synthesis of essential cellular macromolecules required for growth. Accordingly, the FBA problem was formulated as a linear programming (LP) optimization:

(3) $$\mathrm{Maximize}\;Z=c^{T}v$$

subject to:

(4) $$Sv=0,v_{min}\leq v\leq v_{max}$$

where *Z* represents the predicted specific growth rate (h^−1^), and *c* is a coefficient vector with a value of 1 for the biomass reaction and 0 for all others. Solving this optimization yields an optimal flux vector (v*), which satisfies both mass balance and flux constraints while maximizing biomass production. The biomass flux (v*_biomass_) directly corresponds to the predicted specific growth rate (μ) of the organism under defined conditions [58].

#### 4.2.3. Simulation of Nutrient and Prebiotic Conditions

To evaluate metabolic adaptability, each psychobiotic strain was simulated under defined nutrient environments after the reconstruction and refinement of the model. To represent different growth conditions, custom-defined M9 media files (.tsv) supplemented with quercetin were used. BiGG metabolic identifiers were used to parametrize each medium [59]. The defined M9 medium contained ammonium (NH_4_), phosphate (P), sulfate (SO_4_), sodium (Na), potassium (K), chloride (Cl), magnesium (Mg^2+^), calcium (Ca^2+^), and water (H_2_O). In the computational model, quercetin was represented as an extracellular exchange metabolite. The M9 minimal medium was used as a defined reference medium, whereas additional simulations were conducted using quercetin-supplemented M9 minimal medium under microaerophilic conditions. To computationally represent limited oxygen availability, the oxygen uptake rate was constrained to 5 mmol gDW^−1^ h^−1^, following a previously reported constraint-based modelling approach [60]. To define the availability of essential nutrients and quercetin under the simulated conditions, exchange reactions were configured. Accordingly, supplementation of quercetin was evaluated as a computational metabolic perturbation, and the resulting optimal biomass fluxes were interpreted as model-predicted metabolic responses [61]. The complete medium composition and exchange constraints are provided in Supplementary Table S2.

#### 4.2.4. Flux Variability Analysis (FVA)

Flux Variability Analysis (FVA) was performed using COBRApy version 0.24 in Python to evaluate the flexibility of metabolic pathways and to identify the reactions capable of carrying alternative fluxes without compromising optimal growth [45,62]. For each reaction *j*, FVA was formulated as two linear optimization problems to determine the minimum and maximum feasible flux values that maintain the optimal biomass yield (*Z_opt_*). For each model, the biomass reaction was constrained to the optimal biomass flux obtained from the corresponding FBA solution, and the minimum and maximum feasible fluxes of each reaction were subsequently determined under this growth constraint.

(5) $$\mathrm{maximize}/\mathrm{minimize}\;v_{j}$$

subject to:

(6) $$Sv=0,v_{min}\leq v\leq v_{max},c^{T}v=Z_{opt}$$

Reactions exhibiting a flux width of 10^−6^ were classified as rigid, indicating a fixed flux value. The reactions with a wider range of flux values were classified as flexible, reflecting metabolic redundancy. Reactions displaying excessively large flux values were marked as unbound, as they suggested the presence of thermodynamically infeasible cycles (TIC). This analysis provided insights into metabolic redundancy, pathway robustness, and alternative flux routes available to the psychobiotic strains under varying nutrient and prebiotic environments [51].

### 4.3. Network Pharmacology and Gut–Brain Axis Pathway Analysis

#### 4.3.1. Dataset Curation for Selection of Metabolites, Genes, and Quercetin Targets

An extensive literature review was conducted on psychobiotics, depression, gut–brain axis, and microbial metabolomics to identify the gut–brain-associated metabolites. These included short-chain fatty acids (acetate, butyrate, propionate, etc.), tryptophan metabolites (kynurenine, quinolinic acid, etc.), neurotransmitters (GABA, dopamine, epinephrine, norepinephrine, etc.), bile acids (cholic acid, deoxycholic acid, lithocholic acid, etc.), and indole derivatives (indole-3-acetic acid, indole-3-propionic acid, indole-3-lactic acid, etc.). This selection was based on the ability of these metabolites to cross the blood–brain barrier, along with their role in modulating neurotransmitter synthesis, neuroinflammation, and neuroendocrine signaling [11,12,26,27]. Similarly, the host genes associated with depression and gut–brain axis regulation were curated from various databases such as KEGG and Reactome, along with the evidence from literature. Key mechanistic pathways included regulation of the HPA axis (CRH, CRHR1, NR3C1), monoamine signaling (MAO-A, MAO-B, SLC6A4, HTR1A), GABAergic activity (GRIN1, GRM5, SLC1A2, GAD1, GAD2), oxidative stress (NOS, SOD, CAT, MPO), immune responses (TNF, IL1B, IL6, TLR4, etc.), and neuroplasticity (BDNF, NTRK2, CREB1, MAPK) [11,12]. Quercetin targets, both computationally predicted and experimentally validated, were retrieved from Swiss Target Prediction, ChEMBL, and STITCH databases. The targets selected emphasized their involvement in neuroinflammation (NFKB1, MAPK1, JAK2, STAT1, etc.), neurotransmitter modulation, and oxidative stress (SOD, NOS, NFE2L2, CAT, etc.), thereby providing a pharmacological framework linking the neuroprotective activity of quercetin to microbiota-host interaction [14,15].

#### 4.3.2. Pathway Enrichment and Functional Mapping

Metabolite Pathway Analysis. Metabolite pathway analysis was performed using MetaboAnalyst 6.0 (https://www.metaboanalyst.ca/MetaboAnalyst/) (accessed on 7 December 2025). The selected list of metabolites was uploaded, and pathway enrichment analysis was conducted using the Kyoto Encyclopaedia of Genes and Genomes (KEGG) database for homo sapiens as reference (*p* < 0.05, FDR corrected). The significance of the pathway was evaluated by the hypergeometric test, and the impact of metabolites within each pathway was determined by measuring the topology by relative-betweenness centrality value [25,63].

Gene/Protein Pathway Analysis. Gene/protein pathway enrichment analysis was performed using the Reactome Pathway Database (https://reactome.org/) (19 December 2025) through the Reactome Pathway Enrichment Tool v95. The analysis was conducted using Homo sapiens as the reference species, with statistical significance set at *p* < 0.05 and adjusted using FDR correction [64,65].

Network Construction, Analysis, and Integration. Protein-chemical interaction mapping for quercetin was performed using STRING v12.0 (https://string-db.org/) (accessed on 13 January 2026) and STITCH 5.0 (https://stitch.embl.de/) (accessed on 27 January 2026) databases. To ensure biological relevance, Homo sapiens was selected as the reference species. The “Add Chemicals” module of STRING was used to incorporate quercetin along with its protein targets, and an interaction network was generated. The resulting network was integrated with the curated metabolite–gene interactions to visualize the relationships among quercetin, gut-derived metabolites, and host molecular targets. Cytoscape v3.10.4 (https://cytoscape.org/) (accessed on 14 February 2026) was used to construct and visualize an integrated compound-metabolite-gene network comprising three types of interactions, i.e., metabolite-gene, gene-gene, and compound-protein interactions. The Network Analyzer plugin was used to determine the relevant topological values, such as degree, betweenness, and closeness centrality value [24,25,49].

### 4.4. Molecular Docking and Molecular Dynamics (MD)

#### 4.4.1. Screening of Target Proteins

Although several highly connected host genes were identified during network construction using Cytoscape v3.10.4, not all corresponding proteins were suitable for molecular docking due to the absence of well-defined ligand-binding pockets in their three-dimensional structure. Therefore, molecular docking studies were performed only on selected hub target proteins with available high-resolution crystal structures and characterized ligand-binding sites or experimentally reported catalytic or ligand interaction regions. In addition, the proteins that were mainly involved in the metabolism of neurotransmitters [15,66,67], metabolism of flavonoids, microbial hydrolases [68], modulation of inflammatory signaling pathways [69], neurotrophic and intracellular signaling [46,70], and oxidative and endocrine regulation [46,71], as supported by literature evidence, were included to ensure comprehensive pathway coverage. These target proteins play a critical role in mood regulation, gut microbial biotransformation, neuroimmune modulation, antioxidant defense, and modulation of the hypothalamic–pituitary–adrenal (HPA) axis [14]. Table 7 shows a compilation of the key protein targets associated with the regulation of the gut–brain axis, along with their corresponding PDB IDs used for docking analysis.

#### 4.4.2. Protein Preparation

The crystal structures of selected proteins were acquired from the RCSB Protein Data Bank (https://www.rcsb.org/) (accessed on 2 March 2026) in PDB format [72,73]. The protein structures were prepared using UCSF Chimera version 1.19.0. All the water molecules and co-crystallised ligands were trimmed and removed, whereas structurally and functionally relevant cofactors were retained wherever applicable. Then, the structures were energy-minimized [74]. Subsequently, hydrogen atoms were added, and Kollman charges were estimated. The prepared protein structures were converted into .pdbqt format using Autodock Tools version 1.5.7.

#### 4.4.3. Ligand Preparation

The 3D structure of Quercetin (ligand) was retrieved from the PubChem database (https://pubchem.ncbi.nlm.nih.gov/) (accessed on 18 March 2026) in SDF format. The SDF file was converted to PDB format using OpenBabel version 3.1.0. The ligand molecule was then uploaded into Autodock Tools version 1.5.7 and assigned Gasteiger charges, along with the addition of hydrogen atoms and identification and alteration of rotational interactions. The resulting structure was saved in .pdbqt format [75].

#### 4.4.4. Molecular Docking

Molecular docking was performed using Autodock Vina version 1.5.7. The target protein file and ligand structure in .pdbqt format were used as inputs.

For proteins containing a co-crystallised reference ligand, the grid box was centered on the ligand, and its center coordinates were saved in configure.txt format. For proteins lacking a suitable co-crystallised ligand, the docking site was defined on the basis of an experimentally characterized active binding site or ligand-interacting residues as reported in the literature [76,77,78]. The grid box dimensions were chosen to incorporate the relevant binding pocket and to provide sufficient space for ligand sampling. The center coordinates were saved in configure.txt format. Subsequently, the processed protein structures were used for docking studies in Autodock Vina version 1.5.7 [18,75]. The values of the x, y, and z coordinates of the grid center, together with the grid box size, grid spacing, and number of grid points for each of the protein targets, are provided in Supplementary Table S5.

Based on the binding affinity (kcal/mol), the ligand-protein interactions were evaluated, and the results were recorded [73,79]. The resulting interactions were analysed and visualized using Biovia Discovery Studio (v24.1.0.23298) to look for interactions between amino acids [72,75].

#### 4.4.5. Redocking Validation

For the evaluation of the ability of the docking protocol to reproduce experimentally observed ligand-binding orientations, redocking validation was performed for protein targets for which co-crystallised ligand molecules were available. For this, the structure of the ligand was separated from the protein structure, and the resulting structure was saved in .pdbqt format using UCSF Chimera version 1.19.0. This ligand structure was then redocked into its corresponding binding site using the established docking procedure using Autodock Vina version 1.5.7. This predicted redocked pose was then superimposed onto the experimentally determined crystallographic pose, and the root mean square deviation (RMSD) value was calculated using UCSF Chimera version 1.19.0. An RMSD value of less than 2.0 Å was considered indicative of satisfactory reproduction of the experimentally observed binding pose [32,33].

#### 4.4.6. Molecular Dynamics (MD) Simulation

CABS-flex 3.0 web server (https://lcbio.pl/cabsflex3/) (accessed on 21 April 2026) was employed to simulate the dynamic behavior of protein-ligand complexes. Docked protein-ligand complexes were used as input structures for the analysis, with quercetin retained in the complex during the whole simulation. Based on coarse-grained simulations, this web server generates several protein conformations that represent the molecule’s flexibility and structural conformational reorganizations through time. This approach is computationally different from conventional atomistic molecular dynamics simulations with explicit solvent because it uses a coarse-grained simulation approach. Based on simulation results, the root mean square fluctuation (RMSF) was calculated to evaluate the local flexibility of protein residues and overall compactness of the protein. Furthermore, root-mean-square deviation (RMSD) was so as to measure backbone deviations containing in all simulations [73]. Because the coarse-grained model was used for simulation and proteins with different sizes, structures, and conformational flexibility were involved. Therefore, RMSF and RMSD values were mainly interpreted for each protein individually instead of using them as a direct measure of stability across different proteins.

### 4.5. ADMET Profiling

The Simplified Molecular Input Line Entry System (SMILES) of the ligand (quercetin) was extracted from the PubChem database and pkCSM web server (https://biosig.lab.uq.edu.au/pkcsm/prediction) (accessed on 11 May 2026). Furthermore, the SwissADME tool (https://www.swissadme.ch/) (accessed on 19 May 2026) was used to assess the physicochemical, pharmacokinetic (absorption, distribution, metabolism, and excretion), toxicological, and drug-likeness profile of quercetin [18,24].

## 5. Conclusions

This study presents an integrated in silico systems biology approach to investigate the potential therapeutic relevance and molecular interactions of a quercetin–psychobiotic co-delivery system for the treatment of depression via modulation of the gut–brain axis. Genomic and metabolic analyses predicted the presence of key functional genes in the selected psychobiotic strains, including the genes associated with the production of neuroactive metabolites, short-chain fatty acid-mediated microbial interactions, etc. Genome-scale metabolic modeling also predicted significant inter-strain variability across different psychobiotic strains in terms of predicted biomass flux, nutrient utilization, and metabolic adaptability under quercetin-supplemented conditions, enabling the rational selection of high-performing psychobiotic candidates. Strains such as *B. breve* UBBR01 and *L. plantarum* PS128 exhibited higher biomass flux and metabolic adaptability. Also, the results of flux variability analysis (FVA) predicted a balance between the tightly regulated core metabolic pathways and flexible auxiliary pathways involved in the metabolism of carbohydrates, redox balance, and production of short-chain fatty acids.

At the molecular level, quercetin showed predicted strong binding affinity towards multiple protein targets associated with oxidative stress, inflammation, and stress-related pathways. Results of molecular dynamics also corroborated the results of molecular docking, supporting the structural stability of protein-ligand complexes. Favorable pharmacokinetic properties of quercetin were indicated by the results of ADMET profiling, including good intestinal absorption and limited blood–brain barrier permeability. This suggested that quercetin primarily exerts its effects through peripheral, gut-mediated mechanisms.

Overall, these findings provided a computational framework for the selection of suitable psychobiotic strains and predicted that a combination of selected psychobiotics and quercetin could present a promising and mechanistically supported strategy for the modulation of the gut–brain axis. Importantly, this study highlighted the strength of integrated in silico system biology approaches in enabling the rational selection of functional strains and in understanding complex host–microbiome interactions. Our future research will focus on experimental validation of these findings through in vitro and in vivo studies and the development of optimized synbiotic formulations combining selected psychobiotic strains with quercetin for the management of depression by modulation of the gut–brain axis.

## Figures and Tables

**Table 1:** Genomic characteristics of the selected candidate psychobiotic strains.

Candidate Strain	Strain Origin	Genome Size (Mb)	%GC Content	Predicted CDS/Strain	Completeness (%)	Contamination (%)
***B. breve UBBR01***	Unique Biotech Limited	2.3	58.5	1922	99.30	1.79
***L. rhamnosus UBLR58***	Unique Biotech Limited	2.9	46.5	2792	99.06	0.45
***L. helveticus IDCC3801***	Research Laboratories, Ildong Pharmaceutical	2.2	37.0	1851	98.95	0.12
***L. plantarum UBLP40***	Unique Biotech Limited	3.3	44.5	3025	99.35	1.39
***L. plantarum NK151***	Kyung Hee University	3.3	44.5	N/A	68.90	0.41
***L. plantarum 90sk***	Vavilov Institute of General Genetics	3.4	44.5	2969	96.75	1.39
***B. adolescentis 150***	Vavilov Institute of General Genetics	2.3	59.5	1830	96.25	3.39
***L. acidophilus LA5***	Chr. Hansen A/S	2.0	34.5	1800	96.60	0.24
***B. breve M16V***	Swansea University	2.4	58.5	2027	98.71	0.27
***L. plantarum PS128***	Institute of Biomedical Informatics, National Yang Ming University	3.3	44.5	2966	98.69	1.82
***L. helveticus R0052***	Institute Rosell Lallemand Inc.	2.1	37.0	2084	98.85	0.12
***L. plantarum WLPL04***	Nanchang University	3.1	44.5	2882	99.02	1.39

**Table 2:** Major extracellular metabolite uptake and secretion profiles as predicted by the exchange flux analysis.

Strain	Top_Uptakes	Top_Secretions	Interpretation
***B. breve UBBR01***	Ferrous iron, L-Homoserine	Acetate, Carbon dioxide, D-Galactose	Active amino acid and iron-associated metabolism along with secretion of acetate
***L. rhamnosus UBLR58***	Oxygen, Propionaldehyde, L-Homoserine	Acetaldehyde, Carbon dioxide, Glycerol	Adaptable Oxidative and carbohydrate metabolism associated with secretion of aldehyde and glycerol
***L. helveticus IDCC3801***	Hydrogen ion, L-Citrulline, Glycerol-3-phosphate	Carbon dioxide, Ammonium, Inorganic phosphate	Highly regulated energy metabolism and nitrogen turnover
***L. plantarum UBLP40***	Arbutin, Nicotinamide mononucleotide, Phenethylamine	Acetate, Carbon dioxide, D-Galactose	Utilization of aromatic and nucleotide-associated substrates along with secretion of acetate
***L. plantarumNK151***	L-Homoserine, Nicotinamide mononucleotide, Phenethylamine	Acetate, Carbon dioxide, Mannose-1-phosphate	Flexible amino acid and carbon metabolism with secretion of carbohydrate intermediates
***L. plantarum 90sk***	L-Valine, Sulfite, Sucrose	Carbon dioxide, Ammonium, 2-Methylpropanoate	Amino acid catabolism along with sulfur-associated metabolism
***B. adolescentis 150***	Ferrous iron, Hydrogen ion, Nicotinamide mononucleotide	Carbon dioxide, Ferric iron, D-Galactose	Redox-associated metabolic flexibility and carbohydrate metabolism
***L. acidophilus LA5***	Maltose, Water	Carbon dioxide, D-Galactose, Ammonium	Efficient carbohydrate utilization and nitrogen metabolism
***B. breve M16V***	L-Homoserine, Oxygen, Arbutin	Acetate, Carbon dioxide, D-Galactose	Oxidative carbohydrate metabolism along with production of acetate
***L. plantarum PS128***	Alpha-ketoglutarate, Glycerol	Acetate, Carbon dioxide	Dynamic carbon metabolism and redox-balancing
***L. helveticus R0052***	Nicotinamide mononucleotide, Oxygen, Water	Acetate, Hydrogen ion, Ammonium	Production of acetate along with nitrogen turnover
***L. plantarum WLPL04***	L-Homoserine, Nicotinamide mononucleotide	Acetate, Carbon dioxide	Flexible redox-associated pathways and active carbon turnover

**Table 3:** Classification of metabolites on the basis of their functional roles in the modulation of the gut–brain axis.

**Short Chain Fatty Acids** (**SCFAs**)											
Butyrate	Acetate			Propionate		Valerate					Caproate
**Tryptophan and its Derivatives**											
Tryptophan			Kynurenine			Quinolinic acid			Indole derivatives		
**Classical Neurotransmitters**											
GABA	Serotonin			Dopamine		Epinephrine					Norepinephrine
Histamine		Glycine						Glutamate			
**Bile Salts**											
Cholic acid	Deoxycholic acid			Chenodeoxycholic acid		Lithocholic acid					Taurocholic acid
Taurodeoxycholic acid						Ursodeoxycholic acid					
**Microbial Metabolites**											
Succinate			Formate			Trimethylamine			Phenylacetic acid		
Trimethylamine N-oxide (TMAO)						*p*-Cresol sulfate					
**Aromatic Amino Acids Derived Metabolites**											
Phenylalanine						Tyrosine					

**Table 4:** KEGG-based pathway topology analysis with corresponding *p*-values, FDR, and pathway impact scores.

Rank	Pathways	*p*-Value	FDR Value	Impact
1	Butanoate metabolism	5.76 × 10^−5^	0.0046	0.0318
2	Phenylalanine metabolism	0.000182	0.0073	0.3571
3	Tryptophan metabolism	0.000321	0.0086	0.3561
4	Glyoxylate and dicarboxylate metabolism	0.001266	0.0223	0.0833
5	Phenylalanine, tyrosine and tryptophan biosynthesis	0.001394	0.0223	1.0000
6	Tyrosine metabolism	0.003543	0.0472	0.3699

**Table 5:** Significantly enriched metabolic pathways as shown by pathway enrichment analysis.

Rank	Pathways	*p*-Value	FDR Value
1	Glycine, serine and threonine metabolism	6.85 × 10^−10^	5.48 × 10^−8^
2	Phenylalanine, tyrosine and tryptophan biosynthesis	5.34 × 10^−6^	2.14 × 10^−4^
3	Valine, leucine and isoleucine biosynthesis	7.26 × 10^−5^	0.00145
4	Phenylalanine metabolism	7.26 × 10^−5^	0.00145
5	Cysteine and methionine metabolism	0.000435	0.00695
6	Tyrosine metabolism	0.00111	0.0148
7	Pantothenate and CoA biosynthesis	0.00135	0.0155
8	Glyoxylate and dicarboxylate metabolism	0.00492	0.0492

**Table 6:** Significantly enriched biological pathways as shown by Reactome-based pathway analysis.

Rank	Pathways	*p*-Value	FDR Value
1	Metabolism of amino acids and derivatives	2.35 × 10^−6^	1.12 × 10^−4^
2	Tryptophan catabolism	4.21 × 10^−6^	1.88 × 10^−4^
3	Glutathione metabolism	6.15 × 10^−6^	2.30 × 10^−4^
4	Phenylalanine and tyrosine metabolism	1.42 × 10^−5^	3.87 × 10^−4^
5	Neurotransmitter release cycle	2.38 × 10^−5^	4.60 × 10^−4^
6	Glycine, serine and threonine metabolism	3.96 × 10^−5^	5.44 × 10^−4^
7	Citric acid (TCA) cycle	4.35 × 10^−5^	6.12 × 10^−4^
8	Propanoate metabolism	5.26 × 10^−5^	6.85 × 10^−4^
9	Pyruvate metabolism	7.92 × 10^−5^	8.10 × 10^−4^
10	Amino acid transport across plasma membrane	1.23 × 10^−4^	9.75 × 10^−4^

**Table 7:** List of protein targets, their PDB IDs, and associated biological pathways.

Target Protein	PDB ID	Associated Pathway
Monoamine oxidase A (MAO-A)	2Z5X	Neuromodulation
Monoamine oxidase B (MAO-B)	2V5Z	Neuromodulation
Sodium-dependent serotonin transporter (SERT)	5I6X	Neuromodulation
Cytochrome P450 3A4 (CYP3A4)	1TQN	Flavonoid Metabolism
Cytochrome P450 2C9 (CYP2C9)	4NZ2	Flavonoid Metabolism
Cytochrome P450 1A2 (CYP1A2)	2HI4	Flavonoid Metabolism
Flavin-containing monooxygenase 3 (FMO3)	2VQ7	Flavonoid Metabolism
β-Glucuronidase	3HN3	Microbial Degradation
β-Glucuronidase (Bacterial)	3LPF	Microbial Degradation
β-Glucosidase (Bacterial)	5WAB	Microbial Degradation
Bile Salt Hydrolase (BSH)	5HKE	Microbial Degradation
Quercetinase (Bacterial)	1JUH	Microbial Degradation
Azoreductase (Bacterial)	2HPV	Microbial Degradation
Toll-like receptor 4/Myeloid differentiation factor-2 complex (TLR4/MD-2)	3FXI	Inflammatory Signaling
Cyclooxygenase-2 (COX-2)	5IKR	Inflammatory Signaling
Tumor necrosis factor alpha (TNF-α)	2AZ5	Inflammatory Signaling
Interleukin-1 beta (IL-1β)	8C3U	Inflammatory Signaling
Glycogen synthase kinase-3 beta (GSK-3β)	1Q4L	Neurotrophic and Intracellular Signaling
Protein kinase B (Akt/PKB)	1UNQ	Neurotrophic and Intracellular Signaling
Mitogen-activated protein kinase 1 (ERK2/MAPK)	2ERK	Neurotrophic and Intracellular Signaling
Tropomyosin receptor kinase B (TrkB)	4AT4	Neurotrophic and Intracellular Signaling
cAMP-specific 3′,5′-cyclic phosphodiesterase 4 (PDE4)	1XMU	Neurotrophic and Intracellular Signaling
Protein kinase C delta (PKCδ)	1PTR	Neurotrophic and Intracellular Signaling
Superoxide dismutase (SOD1)	2C9V	Oxidative Stress
Catalase (CAT)	1QQW	Oxidative Stress
Glucocorticoid receptor (GR)	4P6X	HPA Axis Modulation
Corticotropin-releasing factor receptor 1 (CRHR1)	4K5Y	HPA Axis Modulation

## Data Availability

The original contributions presented in this study are included in the article/Supplementary Materials. Further inquiries can be directed to the corresponding authors.

## Supplementary Materials

The following supporting information can be downloaded at: https://www.mdpi.com/article/10.3390/ph19091366/s1, Table S1. Characteristics of the reconstructed psychobiotic GEMs. Table S2. Composition and flux constraints of the simulated media. Table S3. Biomass objective reaction of the reconstructed GEMs. Table S4. GEM reconstruction and gap-filling parameters. Table S5: x, y, and z coordinates of the grid center, together with the grid box size, grid spacing, and number of grid points for each of the protein targets. Figure S1. Molecular docking interaction analysis of quercetin with key target proteins (a) Bile Salt Hydrolase, (b) COX 2, (c) TLR4, and (d) IL-1β. Figure S2. Molecular docking interaction analysis of quercetin with key target proteins (e) MAO B, (f) β-glucosidase, (g) TrkB, and (h) β-Glucuronidase 3HN3. Figure S3. Molecular docking interaction analysis of quercetin with key target proteins (i) β-Glucuronidase 3LPF, (j) CYP2C9, (k) GSK3β, and (l) CYP1A2. Figure S4. Molecular docking interaction analysis of quercetin with key target proteins (m) PDE4, (n) Quercetinase, (o) ERK2 MAPK, and (p) Akt/PKB. Figure S5. Molecular docking interaction analysis of quercetin with key target proteins (q) SOD1, (r) MAO A, and (s) PKC-δ.
