# Supplementary material for: Systems Biology and Structure-Based In Silico Evaluations of Quercetin–Psychobiotic Interactions for the Management of Depression
Source: Pharmaceuticals (Basel). 2026 Aug 28;19(9):1366. doi: 10.3390/ph19091366 (PMC13609952; doi:10.3390/ph19091366)
Supplement: Supplementary file 1 [file pharmaceuticals-19-01366-s001.zip › pharmaceuticals-4484019-supplementary.pdf]

SUPPLEMENTARY MATERIALS

Systems Biology and Structure-Based In Silico Evaluations of Quercetin–Psychobiotic Interactions for the Management of Depression

Samridhi Kurl [1], Neeraj Mittal [2], Rajwinder Kaur [2], Balakumar Chandrasekaran [3]* and Gurpreet Kaur [1]*

[1]Department of Pharmaceutical Sciences and Drug Research, Punjabi University Patiala, 147002, Punjab, India.

[2]Chitkara College of Pharmacy, Chitkara University, Chandigarh-Patiala National Highway, Rajpura, 140401, Punjab, India.

[3]Department of Pharmaceutical Sciences, Faculty of Pharmacy, Zarqa University, P.O. Box 2000, Zarqa, 13111, Jordan.

***Correspondence: gurpreetkaur@pbi.ac.in (G.K.); bchandrasekaran@zu.edu.jo (B.C.)

Table S1. Characteristics of the reconstructed psychobiotic GEMs

Psychobiotic Strain	No. of Reactions	No. of Metabolites	Biomass Reaction	Objective Coefficient
B. breve UBBR01	1,949	1,361	Growth	1
B. breve M16V	1,944	1,352	Growth	1
B. adolescentis 150	1,729	1,228	Growth	1
L. acidophilus LA5	1,508	1,016	Growth	1
L. helveticus IDCC3801	1,858	1,218	Growth	1
L. helveticus R0052	1,655	1,146	Growth	1
L. plantarum 90sk	1,344	846	Growth	1
L. plantarumNK151	1,923	1,262	Growth	1
L. plantarum WLPL04	1,883	1,243	Growth	1
L. plantarum PS128	1,894	1,241	Growth	1
L. plantarum UBLP40	1,860	1,227	Growth	1
L. rhamnosus UBLR58	1,867	1,231	Growth	1

Table S2. Composition and flux constraints of the simulated media

Medium Component	BiGG ID	M9 Minimal Medium	Microaerophilic M9 + Quercetin	Flux constraint (mmol gDW^{-1} h^{-1})
Ammonium	NH_4	Yes	Yes	−1000 to 1000
Phosphate	P	Yes	Yes	−1000 to 1000
Sulfate	SO_4	Yes	Yes	−1000 to 1000
Sodium	Na	Yes	Yes	−1000 to 1000
Potassium	K	Yes	Yes	−1000 to 1000
Chloride	Cl	Yes	Yes	−1000 to 1000
Magnesium	Mg^{2+}	Yes	Yes	−1000 to 1000
Calcium	Ca^{2+}	Yes	Yes	−1000 to 1000
Water	H_2O	Yes	Yes	−1000 to 1000
Oxygen	O_2	Yes	Yes	−5 to 0
Quercetin	Quc	No	Yes	−1000 to 1000

Table S3. Biomass objective reaction of the reconstructed GEMs

Parameter	Description
**Reaction ID**	Growth
**Reaction Name**	Biomass Reaction
**Objective Coefficient**	1
**Lower Bound**	0 mmol gDW^{-1} h^{-1}
**Upper Bound**	1000 mmol gDW^{-1} h^{-1}
**No. of Reactants**	53
**No. of Products**	04

The complete stoichiometric equation of the Growth reaction is as follows:

0.000223 10-formyltetrahydrofolate + 0.513689 L-alanine + 0.000223 S-adenosyl-L-methionine + 0.295792 L-arginine + 0.241055 L-asparagine + 0.241055 L-aspartate + 54.124831 ATP + 0.005205 Ca^{2+} + 0.005205 Cl^{-} + 0.000576 CoA + 0.000100 Co^{2+} + 0.133508 CTP + 0.000709 Cu^{2+} + 0.091580 L-cysteine + 0.026166 dATP + 0.027017 dCTP + 0.027017 dGTP + 0.026166 dTTP + 0.000223 FAD + 0.006715 Fe^{2+} + 0.007808 Fe^{3+} + 0.263160 L-glutamine + 0.263160 L-glutamate + 0.612638 glycine + 0.215096 GTP + 48.601527 H_2O + 0.094738 L-histidine + 0.290529 L-isoleucine + 0.195193 K^{+} + 0.450531 L-leucine + 0.343161 L-lysine + 0.153686 L-methionine + 0.008675 Mg^{2+} + 0.000223 5-methyltetrahydrofolate + 0.000691 Mn^{2+} + 0.000100 menaquinol-8 + 0.001831 NAD^{+} + 0.000447 $NADP^{+}$ + 0.185265 L-phenylalanine + 0.221055 L-proline + 0.000223 pyridoxal-5′-phosphate + 0.000223 riboflavin + 0.215792 L-serine + 0.004338 SO_4^{2-} + 0.000223 tetrahydrofolate + 0.000223 thiamine diphosphate + 0.253687 L-threonine + 0.056843 L-tryptophan + 0.137896 L-tyrosine + 0.100000 UDP-N-acetylmuramoyl-L-alanyl-D-glutamyl-meso-diaminopimelate-D-alanyl-D-alanine + 0.144104 UTP + 0.423162 L-valine + 0.000341 Zn^{2+} → 53.950000 ADP + 53.950000 H^{+} + 53.945662 P_i + 0.773903 PP_i.

Table S4. GEM reconstruction and gap-filling parameters

Parameter	Details
**Reconstruction software**	CarveMe v1.6.5
**Reconstruction approach**	Top-down reconstruction
**Candidate reaction pool**	Universal metabolic reaction network
**Gap-filling approach**	Optimization-based gap filling
**Reaction prioritization**	Genomic annotation evidence
**Minimum biomass constraint**	0.1 mmol gDW^{-1} h^{-1}
**Maximum flux (bigM)**	1000 mmol gDW^{-1} h^{-1}
**Activity tolerance**	10^{-9}
**Model curation**	COBRApy v0.24.0
**Final model format**	SBML

Table S5. x, y, and z coordinates of the grid center, together with the grid box size, grid spacing, and number of grid points for each of the protein targets

Target Protein	PDB ID	Crystallographic Ligand/Bound Compounds	Cofactors	Center X	Center Y	Center Z	Box X (Å)	Box Y (Å)	Box Z (Å)	No. of Grid Points	Grid Spacing (Å)
MAO-A	2Z5X	Harmine (HRM)	FAD	40.620	26.910	-14.520	15	15	15	68,291	0.375
MAO-B	2V5Z	Safinamide (SAG)	FAD	51.850	156.43	28.570	15	15	15	68,921	0.375
SERT	5I6X	Paroxetine (8PR)	-	-32.550	-21.300	1.880	15	15	15	68,291	0.375
CYP3A4	1TQN	-	HEM	44.760	-34.370	36.310	15	15	15	68,291	0.375
CYP2C9	4NZ2	2QJ	HEM	-62.580	-45.500	-22.560	15	15	15	68,291	0.375
CYP1A2	2HI4	α-Naphthoflavone (BHF)	HEM	2.500	17.790	20.190	15	15	15	68,291	0.375
FMO3	2VQ7	NADP (NAP)	FAD	-13.860	181.440	-24.200	15	15	15	68,291	0.375
β-Glucuronidase	3HN3	NAG	-	97.823	69.743	158.967	15	15	15	68,921	0.375
β-Glucuronidase (Bacterial)	3LPF	Z77	-	-26.187	5.980	-5.703	15	15	15	68,921	0.375
β-Glucosidase (Bacterial)	5WAB	Glycerol (GOL)	-	-17.093	-45.302	36.649	15	15	15	68,921	0.375
BSH	5HKE	PO_4^{3-} (PO4)	-	98.981	104.061	19.405	15	15	15	68,921	0.375
Quercetinase (Bacterial)	1JUH	NAG	Cu^{2+}	48.484	3.921	81.176	15	15	15	68,921	0.375
Azoreductase (Bacterial)	2HPV	-	FMN	45.782	71.090	23.594	15	15	15	68,921	0.375
TLR4/MD-2	3FXI	FTT	-	28.782	-2.753	5.469	15	15	15	68,921	0.375

COX-2	5IKR	Mefenamic acid (ID8)	COH	38.410	2.510	61.280	15	15	15	68,291	0.375
TNF-α	2AZ5	307	-	-19.163	74.451	33.837	15	15	15	68,921	0.375
IL-1β	8C3U	-	-	-17.290	-11.100	-48.380	15	15	15	68,291	0.375
GSK-3β	1Q4L	679	-	39.063	6.247	34.325	15	15	15	68,921	0.375
Akt/PKB	1UNQ	4IP	-	15.180	24.427	16.345	15	15	15	68,921	0.375
ERK2/MAPK	2ERK	-	-	15.350	40.950	17.650	15	15	15	68,291	0.375
TrkB	4AT4	T6E	-	57.109	29.353	18.960	15	15	15	68,921	0.375
PDE4	1XMU	Roflumilast (ROF)	Zn^{2+}, Mg^{2+}	2.699	1.095	40.941	15	15	15	68,921	0.375
PKCδ	1PTR	13-Acetylphorbol (PRB)	Zn^{2+}	10.902	23.391	24.769	15	15	15	68,921	0.375
SOD1	2C9V	SO_4^{2-}	Zn^{2+}, Cu^{2+}	14.053	-23.443	13.128	15	15	15	68,921	0.375
CAT	1QQW	-	HEM	7.351	41.228	85.750	15	15	15	68,921	0.375
GR	4P6X	Cortisol (HCY)	-	4.086	31.378	-6.763	15	15	15	68,921	0.375
CRHR1	4K5Y	CP-376395 (1Q5)	-	-40.450	-20.820	35.260	15	15	15	68,291	0.375

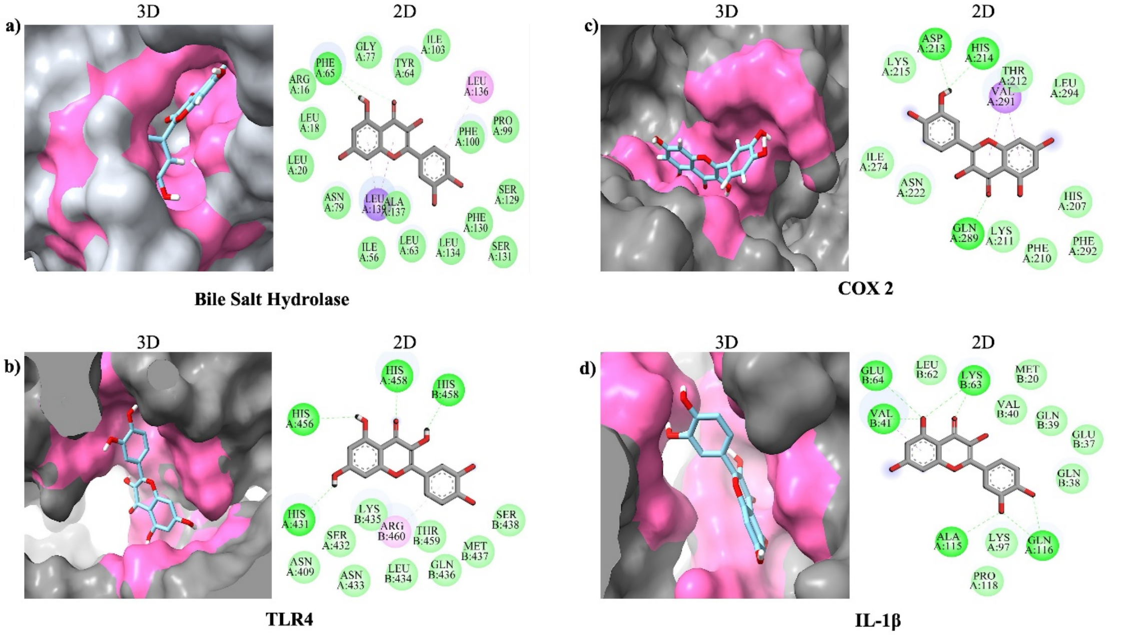

Figure S1. Molecular docking interaction analysis of quercetin with key target proteins (a) Bile Salt Hydrolase, (b) COX 2, (c) TLR4, and (d) IL-1β. The left panels represent the three-dimensional (3D) binding conformations of quercetin within the active binding pockets. In contrast, the right panels depict the corresponding two-dimensional (2D) interaction maps highlighting hydrogen bonding, hydrophobic interactions, π-π stacking, and electrostatic interactions with key amino acid residues.

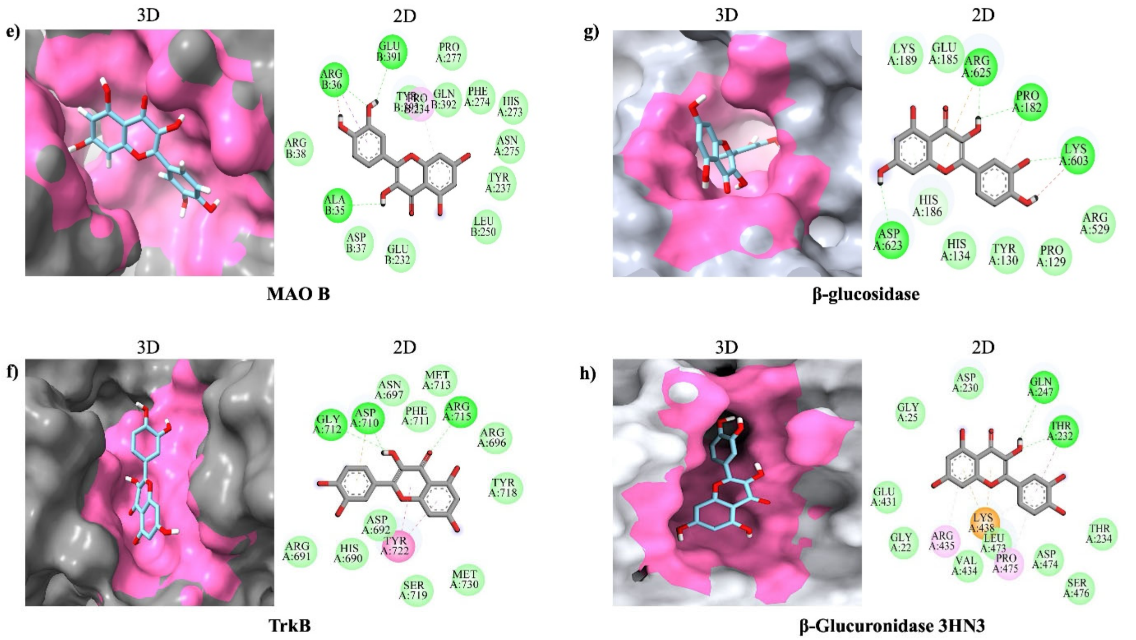

Figure S2. Molecular docking interaction analysis of quercetin with key target proteins (e) MAO B, (f) β-glucosidase, (g) TrkB, and (h) β-Glucuronidase 3HN3. The left panels represent the three-dimensional (3D) binding conformations of quercetin within the active binding pockets. In contrast, the right panels depict the corresponding two-dimensional (2D) interaction maps highlighting hydrogen bonding, hydrophobic interactions, π-π stacking, and electrostatic interactions with key amino acid residues.

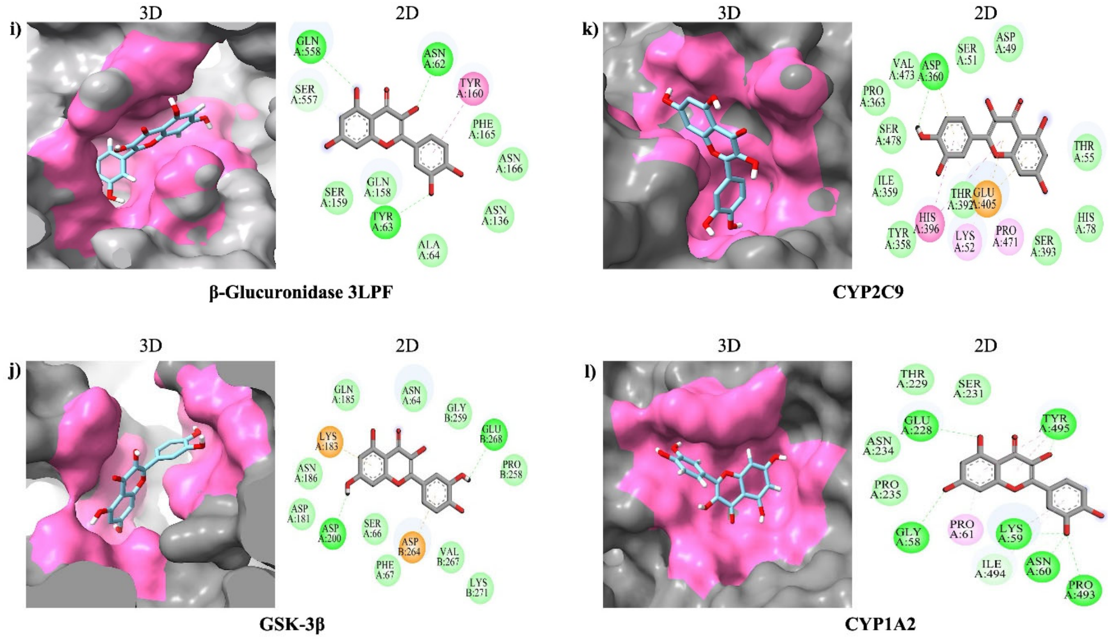

Figure S3. Molecular docking interaction analysis of quercetin with key target proteins (i) β-Glucuronidase 3LPF, (j) CYP2C9, (k) GSK3β, and (l) CYP1A2. The left panels represent the three-dimensional (3D) binding conformations of quercetin within the active binding pockets. In contrast, the right panels depict the corresponding two-dimensional (2D) interaction maps highlighting hydrogen bonding, hydrophobic interactions, π-π stacking, and electrostatic interactions with key amino acid residues.

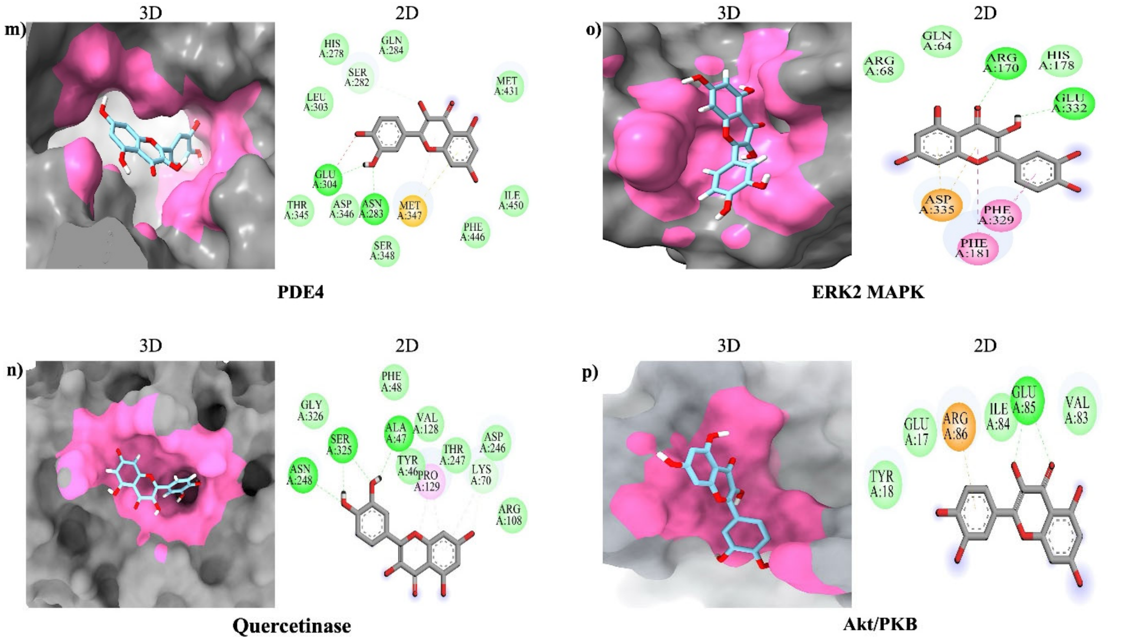

Figure S4. Molecular docking interaction analysis of quercetin with key target proteins (m) PDE4, (n) Quercetinase, (o) ERK2 MAPK, and (p) Akt/PKB. The left panels represent the three-dimensional (3D) binding conformations of quercetin within the active binding pockets. In contrast, the right panels depict the corresponding two-dimensional (2D) interaction maps highlighting hydrogen bonding, hydrophobic interactions, π-π stacking, and electrostatic interactions with key amino acid residues.

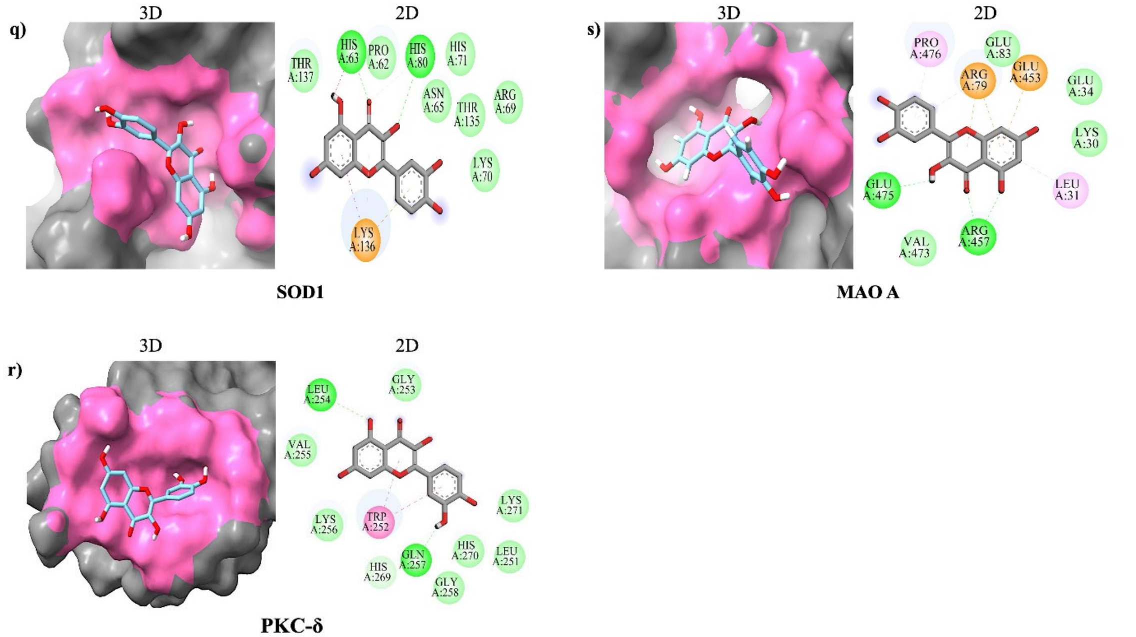

Figure S5. Molecular docking interaction analysis of quercetin with key target proteins (q) SOD1, (r) MAO A, and (s) PKC-δ. The left panels represent the three-dimensional (3D) binding conformations of quercetin within the active binding pockets. In contrast, the right panels depict the corresponding two-dimensional (2D) interaction maps highlighting hydrogen bonding, hydrophobic interactions, π-π stacking, and electro-static interactions with key amino acid residues.
